# Coiled-coil binding of the leucine zipper domains of APOL1 is necessary for the open cation channel conformation

**DOI:** 10.1016/j.jbc.2021.101009

**Published:** 2021-07-29

**Authors:** Charles Schaub, Penny Lee, Alisha Racho-Jansen, Joseph Giovinazzo, Nada Terra, Jayne Raper, Russell Thomson

**Affiliations:** 1 Department of Biological Sciences, Hunter College, City University of New York, New York, New York, USA; 2 The PhD Program in Biochemistry, The Graduate Center of the City University of New York, New York, New York, USA

**Keywords:** APOL1, pH-gated cation channel, planar lipid bilayers, innate immunity, *Trypanosoma brucei*, human African trypanosomiasis (HAT), trypanosome lytic factor (TLF), kidney disease, focal-segmental glomerulosclerosis (FSGS), APOL1-associated nephropathy, APOL1, apolipoprotein L-I, ER, endoplasmic reticulum, MID, membrane-insertion domain, MPB, *N*-(3-maleimidylpropionyl)biocytin, MTSET, [2-(trimethylammonium)ethyl] methanethiosulfonate, bromide, SCAM, substituted-cysteine accessibility method, SRA, serum resistance-associated protein, TLF, trypanosome lytic factor

## Abstract

Apolipoprotein L-I (APOL1) is a channel-forming effector of innate immunity. The common human APOL1 variant G0 provides protection against infection with certain *Trypanosoma* and *Leishmania* parasite species, but it cannot protect against the trypanosomes responsible for human African trypanosomiasis. Human APOL1 variants G1 and G2 protect against human-infective trypanosomes but also confer a higher risk of developing chronic kidney disease. Trypanosome-killing activity is dependent on the ability of APOL1 to insert into membranes at acidic pH and form pH-gated cation channels. We previously mapped the channel’s pore-lining region to the C-terminal domain (residues 332–398) and identified a membrane-insertion domain (MID, residues 177–228) that facilitates acidic pH-dependent membrane insertion. In this article, we further investigate structural determinants of cation channel formation by APOL1. Using a combination of site-directed mutagenesis and targeted chemical modification, our data indicate that the C-terminal heptad-repeat sequence (residues 368–395) is a *bona fide* leucine zipper domain (ZIP) that is required for cation channel formation as well as lysis of trypanosomes and mammalian cells. Using protein-wide cysteine-scanning mutagenesis, coupled with the substituted cysteine accessibility method, we determined that, in the open channel state, both the N-terminal domain and the C-terminal ZIP domain are exposed on the intralumenal/extracellular side of the membrane and provide evidence that each APOL1 monomer contributes four transmembrane domains to the open cation channel conformation. Based on these data, we propose an oligomeric topology model in which the open APOL1 cation channel is assembled from the coiled-coil association of C-terminal ZIP domains.

Apolipoprotein L-I (APOL1) is a cytolytic, channel-forming protein of the primate innate immune system. Broadly expressed in endothelial and epithelial cell types, APOL1 is targeted to the lumen of the endoplasmic reticulum (ER) by an N-terminal signal peptide (1, 2). Most APOL1 is retained in the ER, associated with the lumenal face of the ER membrane, although a minority will proceed along the secretory pathway, reaching the cell surface as a plasma membrane-associated protein (3). In a process that is mostly restricted to hepatocytes, APOL1 can also be secreted into blood plasma, as a subclass of high-density lipoprotein complexes called trypanosome lytic factors 1 and 2 (TLF 1 and TLF 2) (4), which provide protection against infection by many protozoan parasites of the genera *Trypanosoma* and *Leishmania* (5, 6, 7, 8).

In order to provide immunity against trypanosomes, TLF carrying APOL1 must first be endocytosed into acidic parasite endosomes. Purified recombinant APOL1 is taken up by fluid phase endocytosis *in vitro*, resulting in a delayed form of trypanosome killing, whereas primate TLFs have evolved ligands that bind to parasite surface proteins and promote rapid endocytosis of the APOL1-containing complex (9). Thus, in addition to APOL1, and the structural scaffold protein apolipoprotein A-I (APOA1), circulating TLF1 and TLF2 also contain the hemoglobin-binding protein haptoglobin-related protein (Hpr), which binds to a parasite haptoglobin–hemoglobin receptor and promotes endocytosis of the TLF1 complex (10). Alternatively, in the case of TLF2, Hpr is masked by a molecule of polyreactive immunoglobulin M (IgM), which binds to the parasite’s variant surface glycoprotein and allows for rapid endocytosis of TLF2 *via* a trypanosome antibody clearance pathway (11).

Once inside the parasite's endosome, APOL1 inserts into lipid membranes at acidic pH, forming closed-state pH-gated cation channels. Upon recycling to the plasma membrane, APOL1 channels open at neutral pH, leading to dysregulated ion flux, followed by osmotic lysis of the parasite (12, 13, 14). Mitochondrial dysfunction was also implicated in trypanosome killing by APOL1 (15), although this may be a downstream effect of plasma membrane depolarization and ion flux (16). The two trypanosome subspecies that cause disease in humans, *T. b. rhodesiense* in east Africa, and *T. b. gambiense* in west Africa, have evolved resistance to APOL1-mediated lysis. In the case of *T. b. rhodesiense*, resistance is due to the serum resistance-associated protein (SRA), which binds to APOL1 in endosomes and prevents channel formation at acidic pH (14, 17). Alternatively, *T. b. gambiense* resistance to TLF involves reduced TLF1 uptake, due to mutations in the haptoglobin–hemoglobin receptor (18), as well as reduced susceptibility to endocytosed APOL1, which depends on the *T. b. gambiense*-specific glycoprotein (TgsGP, (19)).

In turn, two human APOL1 variants arose in Africa (4000–10,000 years ago), which can kill human-infective trypanosomes and are associated with protection against human African trypanosomiasis in the heterozygous or homozygous genotype (20, 21). However, in an example of heterozygote advantage, the cost of protection from two copies of the variant *APOL1* genes is an increased lifetime risk of developing chronic kidney disease. Individuals with two APOL1 renal risk variants (G1 and/or G2) have a 5% to 10% lifetime risk of developing glomerulosclerosis, and this increases to 50% when combined with human immunodeficiency virus 1 infection (22). It is well established that interferons induce upregulation of *APOL1* gene expression (as well as other *APOL* family genes), leading to the suggestion that the interferon response to viral infection triggers the development of kidney disease *via* the upregulation of APOL1 G1 or G2 variants. This was supported by the association of APOL1 renal risk variants with kidney disease triggered by interferon therapy (23), and more recently COVID-19 infection (24). Thus, APOL1 G1 and G2 are apparently not loss-of-function variants. Indeed, a human family found to lack functional APOL1 genes (due to frameshift mutations in both alleles) (25, 26) had no obvious kidney dysfunction, and chimpanzees, our closest primate relatives, as well as most other primates and all other mammals, lack the APOL1 gene altogether.

APOL1-induced nephropathy likely results from dose-dependent gain of cytotoxic function, which requires dosing of transcripts from two copies of the *APOL1* G1/G2 alleles, combined with interferon-induced upregulation of their transcripts in podocytes (27). Although APOL1 is intrinsically cytotoxic when overproduced in mammalian cells, the APOL1 G1 and G2 proteins were more cytotoxic than APOL1 G0 when produced at levels comparable with those of interferon-stimulated podocytes (28, 29, 30). Cytotoxicity in mammalian cells was dependent on release of APOL1 from the ER, into an acidic intracellular compartment such as the *trans* Golgi and/or secretory vesicles; APOL1 was then detected at the plasma membrane as cells swelled and cytotoxicity was observed (30). In the same study, cytotoxicity was inhibited by replacement of extracellular sodium with choline and the reduction of the extracellular calcium concentration. In another study, patch clamping analysis confirmed that overexpression of APOL1 in mammalian cells resulted in a pH-dependent cation conductance across the plasma membrane (31). These data suggest a basic mode of cytotoxicity that is common to trypanosomes and mammalian cells, involving APOL1 activation (closed state ion channel formation) in an acidic intracellular compartment, followed by the formation of open APOL1 cation channels upon neutralization in the plasma membrane.

Despite relevance to both parasitic disease and kidney disease, direct structural characterization of the APOL1 channel has been challenging, likely due to the possible existence of multiple lipid-bound conformations and oligomerization states. These include high-density lipoprotein–bound, membrane-associated, membrane-inserted, and closed-state and open channel-forming conformations (16, 32). In addition, structure–function studies have been hampered by a lack of homology to known channel forming structures, although unfortunately the N-terminal portion of the APOL1 protein (residues 60–235) was initially dubbed the “pore-forming domain” based on a tenuous analogy to bacterial pore-forming colicins (33). Recent crystal and NMR structures of the N-terminal domain (residues 65-168) reveal a four helical bundle with a fifth long flexible helix, which is hypothesized to move and allow the interaction of a leucine zipper (intra or inter) with the four helical bundle (34).

Recently, we began to characterize the formation of cation channels by recombinant APOL1 in planar lipid bilayers. Membrane insertion of APOL1 requires acidic pH, whereupon it forms cation channels that are gated by *cis* pH, *i.e.*, the membrane side equivalent to the lumenal/extracellular compartments (14, 30). Most recently, we used interspecies APOL1 chimeras to identify a putative central hairpin of transmembrane helices that is required for pH-dependent membrane insertion (membrane-insertion domain; MID, residues 177–228) and mapped the pore-lining region of the channel to the C-terminal domain (16). Furthermore, we identified aspartate 348 (D348) as a critical determinant of pH-dependent cation channel conductance, with the nearby residues Tyr351 and Glu355 having an accessory role in pH gating. These residues align along one face of the putative pore-lining alpha-helix near the C-terminus (residues 335–356) (16).

Early studies showed that the adjacent leucine zipper domain at the extreme C-terminus (Fig. 1; residues 368–395) is also critical for function, with gross deletions and mutations in this region resulting in a loss of trypanolytic activity (6, 14, 35). Leucine zippers are a particular form of parallel coiled-coil dimers, wherein heptad-repeat leucines (*d*-position) pack alongside each other at the dimer interface, nestling into a hole formed by four residues on the opposing helix (knobs into holes packing; Fig. 1*B*) (36). These *d*-position leucine residues are conserved throughout the APOL protein family suggesting an important functional role (37). We have hypothesized that the APOL1 C-terminal heptad-repeat leucines mediate APOL1 oligomerization, bringing together the adjacent pore-lining regions from at least two APOL1 monomers to form a channel (16). However, the potential role of the heptad-repeat leucine residues in oligomerization, channel formation, and trypanosome lysis has yet to be assessed biochemically.

**Figure 1:**
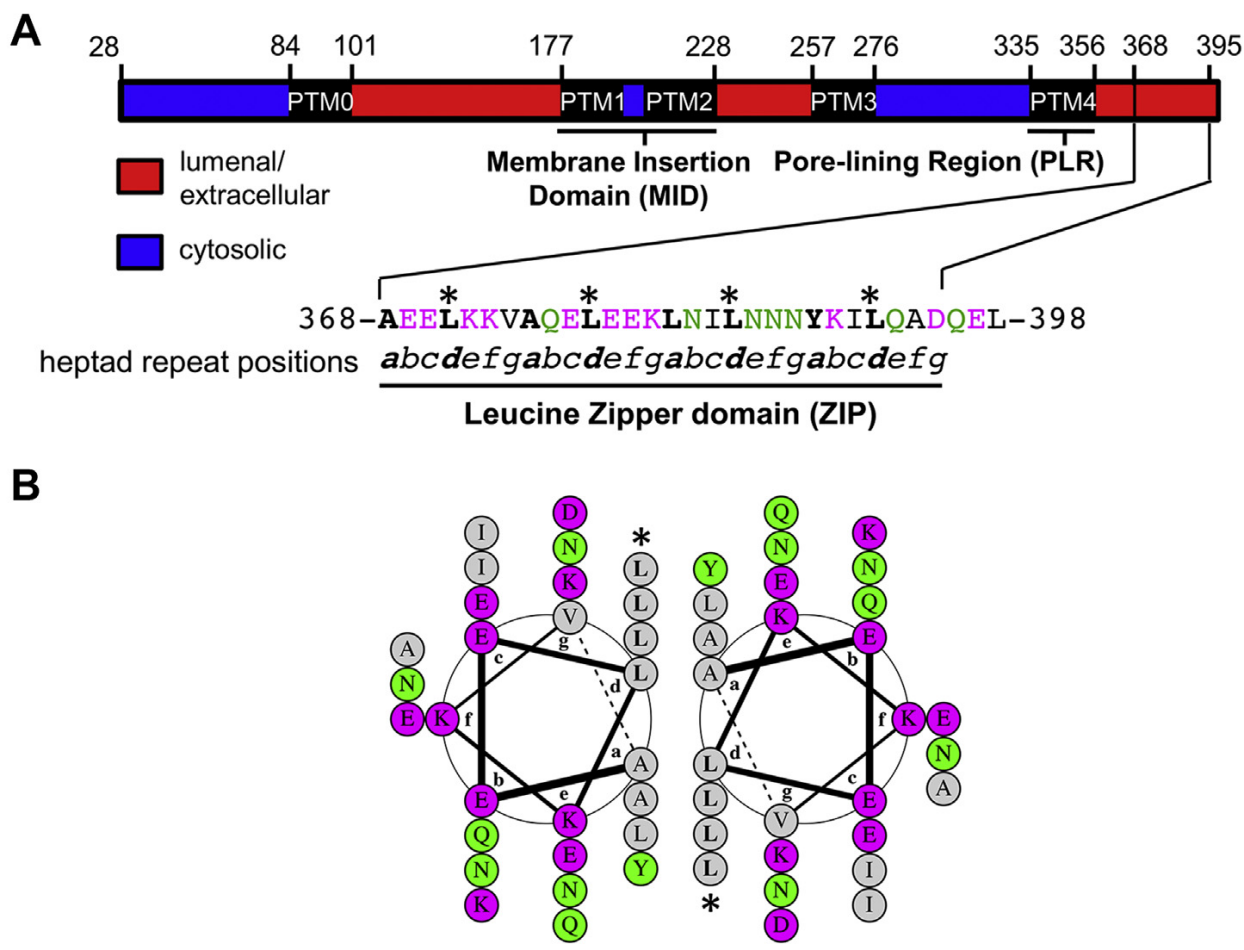
**APOL1 has a leucine zipper domain at the C terminus.***A*, *top*, domain structure of APOL1 showing putative transmembrane domains (PTM 0–PTM 4; see also Table S1) and topology based on *in silico* prediction. *Bottom*, the C-terminal 30 amino acid residues of APOL1 constitutes a leucine zipper domain, which contains both a leucine (371, 378, 385, 392) and a hydrophobic residue (368, 375, 382, 389) every seventh amino acid. Within this heptad repeat pattern (positions a–g), the hydrophobic residue occupies position *a* and the leucine residue occupies position *d* (indicated by an *asterisk*). Hydrophobic residues are shown in *black*, charged residues in *magenta*, and uncharged polar residues in *green*. *B*, DrawCoil 1.0 (58) helical wheel diagram of the APOL1 C-terminal leucine zipper domain. The *a* and *d*-position residues are expected to constitute the hydrophobic face of an amphipathic alpha-helix that is suited to form a leucine zipper type coiled-coil homodimer. Leucine zipper dimers are stabilized by the packing of *d*-position leucine side chains of one helix into the hole formed by four hydrophobic residues on the opposing helix, known as “knobs-into-holes” packing. Charged residues at the exterior can further stabilize dimer formation *via* electrostatic interactions.

In addition, the overall topology of the APOL1 channel structure remains unclear, with secondary structure prediction software identifying anywhere from 2 to 5 transmembrane helices per APOL1 monomer (Table S1). Our initial analysis, which concerns only the open channel state following APOL1 incorporation into planar lipid bilayers, suggests that the open channel contains at least four transmembrane regions per APOL1 monomer (16). This would situate the *cis*-pH sensing residue, E355 on the *cis* side (the side equivalent to the lumenal low-pH/extracellular neutral-pH compartments) of the membrane, at the C-terminal end of the pore-lining alpha-helix. A different conclusion was reached by probing accessibility of APOL1 epitopes to monoclonal antibody binding after APOL1 expression in podocytes (32). According to this analysis, APOL1 at the cell surface contains only a centrally located hairpin of transmembrane helices (the MID), and a possible re-entrant pore-loop near the C terminus, instead of a fully transmembrane pore-lining helix. However, it should be noted that, in contrast to the planar lipid bilayer study, wherein only the open ion channel state could be assessed, the results of the antibody study reflect a pooled average of all conformational states present on the plasma membrane, not just the open channel conformation. Additional evidence is clearly needed to correctly identify the number and location of APOL1 transmembrane regions present in the open channel state.

In this study, we systematically substituted the heptad-repeat leucine residues, alone and in combination, to examine their role in APOL1 oligomerization, cation channel formation and trypanosome lysis. We then used cysteine-scanning mutagenesis across the entire APOL1 sequence, combined with the substituted-cysteine accessibility method (SCAM), to probe the topology of the open APOL1 channel in planar lipid bilayers. Our data suggest that APOL1 channel formation requires leucine zipper–mediated oligomerization between the C-termini of APOL1 monomers, with each monomer contributing four transmembrane domains to the structure of the open channel state.

## Results

### APOL1 contains a leucine zipper motif at the C terminus

The C-terminus of APOL1 is integral for protein function, because truncations result in the loss of trypanolytic and cation channel activity (6, 14, 35). Moreover, the C-terminus contains a leucine zipper motif, which is C-terminal to the pore-lining region of APOL1 (16). Leucine zipper (ZIP) motifs are distinguished by a leucine residue positioned every seventh amino acid, with similarly spaced hydrophobic residues (Fig. 1*A*). The motif assumes an alpha-helical conformation, with leucines aligning on one face of the helix in the “*d*” position and hydrophobic residues occupying the nearby “*a*” position (Fig. 1*B*). Of note, two monomers of a protein containing ZIPs are able to form a dimer, with the branched chain leucine residues (*d*) of one helix filling the holes formed by four hydrophobic residues on the opposing helix (knobs-into-holes packing; (36)). This interaction between the helices’ hydrophobic pockets is stabilized by van der Waals forces (38). Further stability can be conferred to the dimer through interhelical salt bridges (electrostatic interactions) between charged residues at “*g*” and “*e*” positions ((39), Fig. 1*B*).

### The C-terminal leucine zipper motif is critical for trypanolytic activity

**Figure 2:**
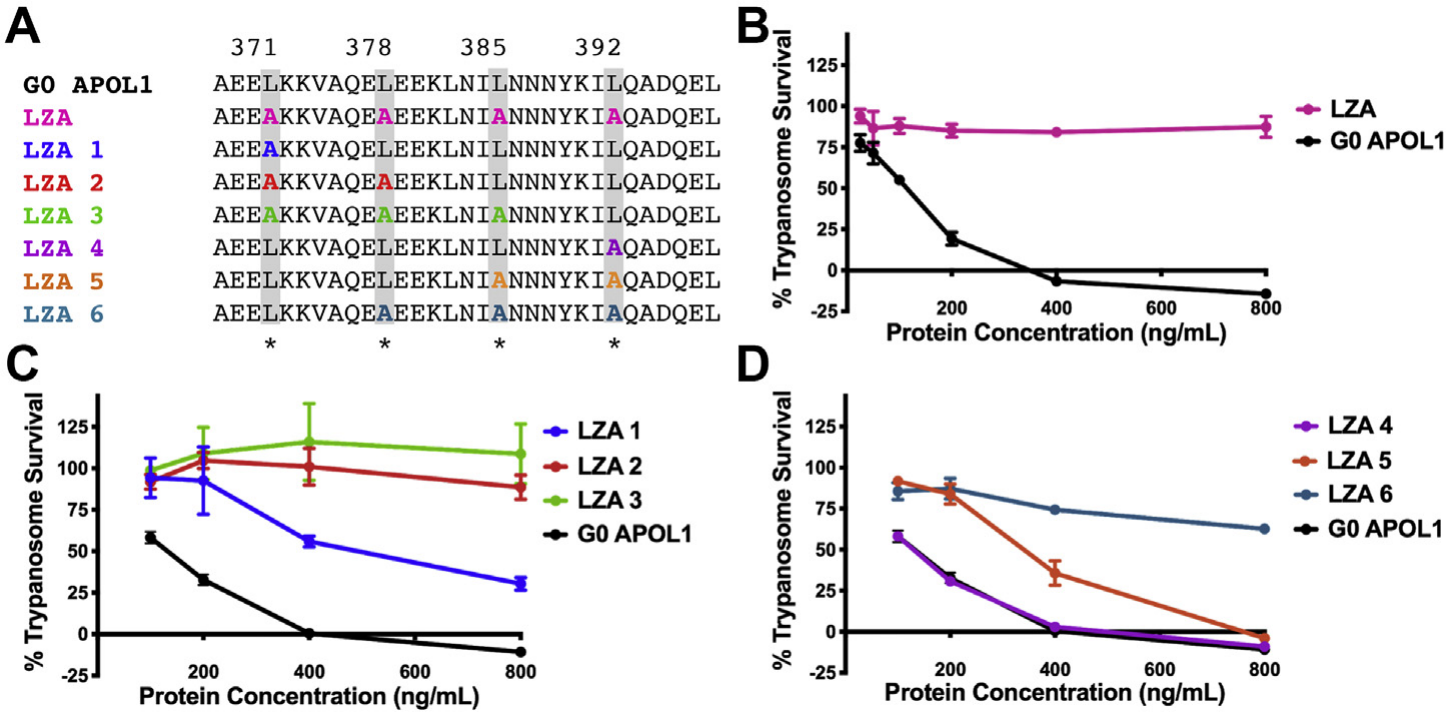
**Functional effects of sequential alanine substitutions in the C-terminal leucine zipper domain of APOL1.***A*, leucine zipper domain amino acid sequences of G0 APOL1 and the purified APOL1 mutants, in which the indicated *d*-position leucines (*shaded gray*) were replaced by alanines, either entirely (LZA) or sequentially (LZA1–6). *B*–*D*, trypanosome lysis assays. Plotted is mean trypanosome survival ±SD of three replicate wells, after 24 h incubation with the indicated concentrations of G0 APOL1 or APOL1 LZA mutants. *B*, APOL1 LZA (L371A/L378A/L385A/L392A) was nontrypanolytic. *C* and *D*, we observed a positive correlation between the number of substituted alanines and loss of trypanolytic activity, with substitutions at the N-terminal end of the leucine zipper domain (LZA1–3) being more detrimental than those at the C-terminal end (LZA4–6). Of note, the single substitution of the most N-terminal leucine (LZA1) was enough to reduce trypanolytic activity compared with APOL1 G0, whereas substitution of only the most C-terminal leucine (LZA4) had no effect. The data plotted are from a single representative experiment (of three independent experiments), wherein all APOL1 LZA1 to 6 proteins were tested in the same 96-well plate.

To better understand the role of the C-terminal ZIP motif in regards to trypanolytic and cation channel activity, we generated recombinant APOL1 proteins with targeted point mutations within the ZIP (Fig. 2*A*). The first mutant APOL1 protein purified and tested, APOL1 LZA, had all four *d* position leucines within the C-terminal heptad repeat substituted for alanine. We chose alanine to replace the branched-chain leucine residues owing to a shorter R-group yet similar hydrophobic properties. There was no evidence of trypanolytic activity *in vitro* at any concentration tested, in contrast to APOL1 G0 tested within the same experiment (Fig. 2*B*). Similarly, APOL1 LZA was unable to cause significant cell death when transiently overexpressed in HEK293 cells, whereas APOL1 G0 was cytotoxic (Fig. S1). Loss of function of APOL1 LZA indicates that the C-terminus contains a ZIP domain.

To identify which, if not all, of the leucine residues are vital for trypanolysis, we generated sequential leucine to alanine mutants starting from both the N-terminal and C-terminal ends of the ZIP domain (Fig. 2*A*). We tested if each of these alanine-substituted APOL1 proteins lyse trypanosomes *in vitro* (Fig. 2, *C* and *D*). The APOL1 LZA 1, with a single substitution at the N-terminal end of the ZIP domain (L371A), had less trypanolytic activity, with an IC_50_ of approximately 600 ng/ml (14.3 nM) compared with 125 ng/ml (2.9 nM) for APOL1 G0. Additional substitutions of leucines L378 and L385 resulted in a complete loss of trypanolytic activity (APOL1 LZA 2 and APOL1 LZA 3) (Fig. 2*C*). In contrast, the APOL1 LZA 4 (L392A) at the C-terminal end of the ZIP domain displayed trypanolytic activity nearly identical to APOL1 G0 (Fig. 2*D*). However, APOL1 LZA 5 (IC_50_ = 350 ng/ml) and APOL1 LZA 6 (IC_50_ > 800 ng/ml) exhibited substantially less trypanolytic activity than APOL1 G0 (Fig. 2*D*). Taken together, these data strongly suggest that, within the ZIP domain, the N-terminal leucines (L371 and L378) are vital for trypanolytic activity, whereas L385 and L392 are relatively interchangeable. Furthermore, the data indicate that cooperation between the *d* position leucines is crucial for APOL1 trypanolytic activity.

### The C-terminal leucine zipper is required for channel formation in planar lipid bilayers

**Figure 3:**
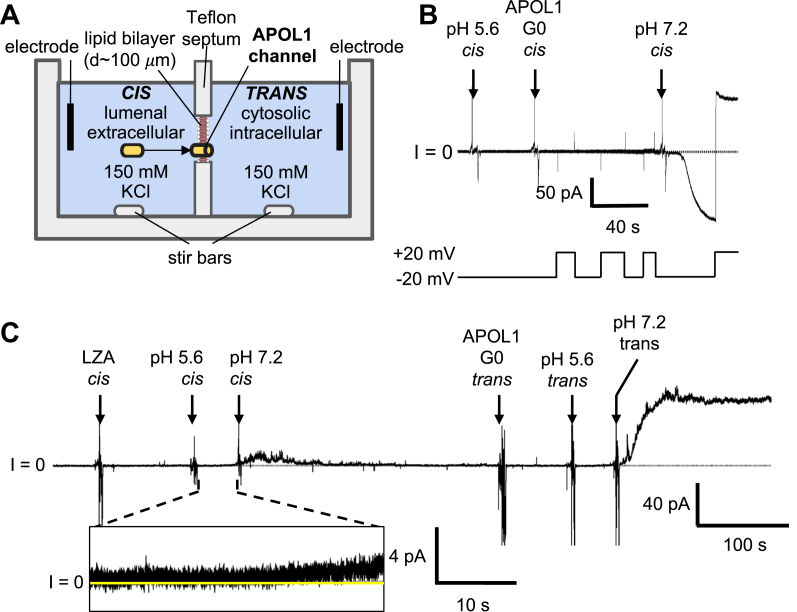
**APOL1 LZA lacks channel forming activity.***A*, schematic of the planar lipid bilayer system. *B*, effect of APOL1 G0 on bilayer current as the voltage was alternated between ±20 mV. The *cis* side was first adjusted to pH 5.6 and then APOL1 G0 was added. The current magnitude increased slightly and then the pH was neutralized, resulting in a large increase in the current. *C*, a constant voltage of +20 mV was applied, and the current was recorded. With symmetric pH 7.2 conditions, 150 ng of APOL1 LZA was added to the *cis* side of the bilayer, which was then acidified to pH 5.6. This was followed by a slight increase in the current (*inset*, expanded 4-pA current scale), indicating protein insertion into the bilayer. When the *cis* side was then neutralized to pH 7.2, there was a brief increase in the current magnitude and noise, but this soon subsided and the current returned to near zero. In contrast, addition of the same concentration of APOL1 G0 to the *trans* side resulted in the usual increase in current magnitude following *trans* acidification and reneutralization. The data shown are representative of at least three independent experiments.

In order to determine if loss of APOL1 LZA trypanolytic activity was correlated to cation channel-forming activity, we utilized the planar lipid bilayer system (Fig. 3*A*). This is an exquisitely sensitive measurement of ion channel activity that can detect single channel activity or the macroscopic activity due to many thousands of channels, recording the ions that move through the channel as current. Figure 3*B* shows the typical macroscopic current response after addition of APOL1 G0 to the *cis* side (the side equivalent to the lumenal low-pH/extracellular neutral-pH compartments) and following the acidification and neutralization of the *cis* pH to mimic endocytic recycling in trypanosomes (Fig. S2). Note that pH neutralization causes an ∼200-fold increase in current owing to the opening of pH-gated channels as previously reported (14, 16). In a separate experiment, APOL1 LZA was added to the *cis* chamber, with symmetrical pH 7.2 on both sides (Fig. 3*C*). The result of subsequent acidification of the *cis* chamber to pH 5.6, to mimic endocytosis of APOL1 and delivery to the acidified endosome within the trypanosome (Fig. S2), was a slight increase in macroscopic conductance, which we interpret as the APOL1 LZA protein inserting into the bilayer (membrane inserted prechannel). After approximately 30 s, the *cis* chamber was neutralized to pH 7.2, to mimic the recycling of the endosome to the plasma membrane in trypanosomes (Fig. S2), and in order to assess if there was any detectable pH-gated opening of the ion channel. Initially, the membrane displayed slight instability, followed by current stabilization with no overall increase in conductance (Fig. 3*C*). To confirm that we indeed had a stable bilayer, an equivalent concentration of APOL1 G0 was then added to the *trans* side. The pH procedure was repeated in the *trans* compartment, which was followed by the expected increase in conductance (ion flow) observed upon pH neutralization (Fig. 3*C*). Thus, substitution of the four heptad-repeat leucines in the C-terminal ZIP domain results in loss of APOL1 open ion channel-forming activity.

### Targeted modification of the leucine zipper hydrophobic pocket prevents channel formation by APOL1

**Figure 4:**
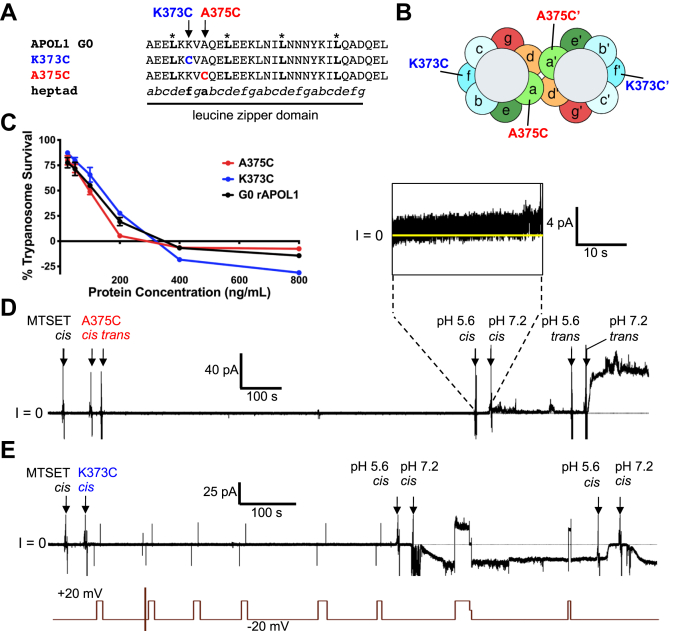
**Targeted cysteine substitution and the effect of conjugation with MTSET.***A*, the C-terminal amino acid sequences of APOL1 A375C and K373C proteins compared with APOL1 G0. *B*, helical wheel projections showing the location of the A375C (*a*-position) and K373C (*f*-position) within the putative leucine zipper dimer. Conjugation of positively charged MTSET (278 Da) to A375C at the dimer interface is expected to prevent dimer formation owing to steric interference and charge repulsion, whereas conjugation to the peripheral K373C (*f*-position) is expected to allow dimer formation. *C*, i*n vitro* 24-h trypanolytic assay. Plotted is mean trypanosome survival ±SD APOL1 A375C and K373C proteins showed no loss of lytic activity compared with APOL1 G0. *D* and *E*, effect of preincubation with MTSET on channel formation by APOL1 K373C and A375C proteins in planar lipid bilayers. The data shown are representative of three independent experiments. *D*, the current was recorded as the voltage was held at +20 mV. MTSET, 1 mM, was preadded to the *cis* chamber and then ~175 ng/4 nM APOL1 A375C (*a*-position) was added to *both* the *cis* and the *trans* chambers. After a 10-min reaction time, the *cis* chamber was acidified for ~30 s and then neutralized, but there was little evidence of channel opening. (*Inset*: above, a slight increase in current at pH 5.6 indicates protein insertion into the bilayer.) Conversely, when the *trans* chamber (which contained APOL1 A375C but no MTSET) was then acidified and neutralized, the usual increase in channel conductance occurred. *E*, the above experiment was similarly performed with APOL1 K373C (*f*-position) and a 250,000-fold molar excess of MTSET. The current was recorded (*upper trace*) as the voltage was manipulated (*lower trace*). Channel formation occurred normally upon acidification and neutralization (a downward deflection in the current indicates channel opening at −20 mV; the delayed current response to acid and base additions was due to a stirring delay). MTSET, [2-(trimethylammonium)ethyl] methanethiosulfonate.

To further investigate the C-terminal ZIP domain, we wanted to generate and purify functional APOL1 proteins without disrupting the heptad-repeat leucine residues (*d* position). We therefore focused on the *a* position on the hydrophobic face and the “*f*” position on the hydrophilic face of the amphipathic helix. Individual cysteines were substituted into the hydrophobic dimer interface (A375C, *a* position) and hydrophilic peripheral face (K373C, *f* position) of the ZIP domain (Fig. 4, *A* and *B*). We were able to specifically target these cysteines with thiol-reactive groups because APOL1 does not contain any endogenous cysteines. Conjugation of a large charged molecule to A375C (*a* position) at the dimer interface was expected to prevent dimer formation owing to steric interference and charge repulsion, whereas conjugation to the peripheral K373C (*f* position) would not prevent dimer formation. Fortunately, the APOL1 A375C and APOL1 K373C proteins had near identical trypanolytic activity against trypanosomes as APOL1 G0 *in vitro*, indicating that the individual cysteine substitutions were well tolerated (Fig. 4*C*).

Having established the functionality of APOL1 A375C and APOL1 K373C *in vitro*, we again turned to the planar bilayer system to observe channel activity. Considering that leucine zipper dimers are stabilized through interactions in the hydrophobic pocket, we examined if addition of the positively charged thiol-reactive molecule [2-(trimethylammonium)ethyl] methanethiosulfonate, bromide (MTSET, 278.2 Da) would have any measurable effect on channel formation by the cysteine-substituted proteins (see Experimental procedures section). A 250,000-fold molar excess of MTSET (compared with APOL1) was added into the *cis* side of the bilayer followed by the addition of APOL1 A375C at pH 7.2 for 10 min to allow for the rapid and very specific covalent (disulfide) modification of the cysteine (Fig. 4*D*). The chamber was then acidified (pH 5.6) to facilitate insertion of protein into the bilayer, which was evident by the modest ∼1-pA increase in current. However, upon reneutralization of the pH to 7.2, no obvious change in conductance was observed (Fig. 4*D*), suggesting APOL1 A375C-MTSET was unable to form pH-gated ion channels. Conversely, APOL1 A375C added to the *trans* side of the same bilayer and put through the same pH procedure without the addition of MTSET produced the usual conductance increase upon reneutralization (Fig. 4*D*). This confirmed that the alanine to cysteine substitution itself did not substantially alter channel-forming activity and the observed loss of activity by the cysteine modified APOL1 A375C-MTSET was not due to a compromised bilayer.

Although MTSET addition to the APOL1 A375C protein greatly diminished channel activity, it remained to be seen if there was a similar effect with the APOL1 K373C. Thus, a similar bilayer experiment was performed, and APOL1 K373C was incubated with a 250,000-fold molar excess of MTSET for 10 min prior to acidification of the *cis* chamber (Fig. 4*E*). APOL1 K373C was able to insert into the membrane and subsequent neutralization caused an increase in channel conductance as observed with APOL1 G0 (Fig. 4*E*). The channels also responded accordingly when the pH in the chamber was altered between neutral pH and acidic pH, thus indicating that the MTSET had no effect on the ability of APOL1 K373C to form pH-gated channels (that is open and close in a pH-dependent manner). We conclude that the effect of MTSET on APOL1 A375C is due to the cysteine’s position within the hydrophobic pocket, consistent with the predicted elimination of a coiled-coil dimer by MTSET-induced electrostatic repulsion and/or steric hindrance.

### Acidification of the C-terminus drives APOL1 dimerization

**Figure 5:**
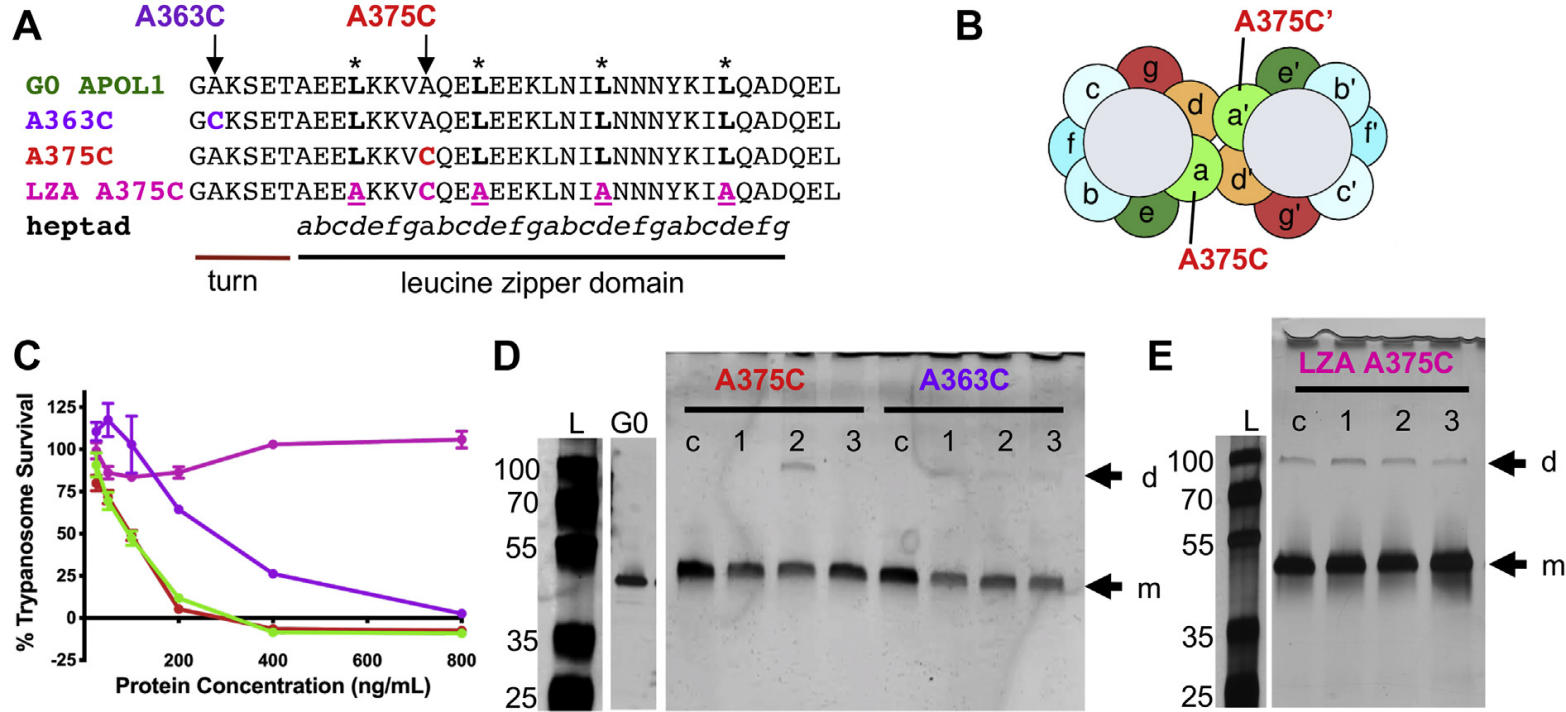
**APOL1 A375C forms pH-dependent disulfide dimers.***A*, C-terminal amino acid sequence alignment of APOL1 A363C (*purple*), A375C (*red*) and LZA A375C (*pink*) mutant proteins compared with APOL1 G0. Note that, unlike the A375C substitution (*a*-position), the A363C substitution is outside of the leucine zipper domain. *B*, helical wheel projection of the leucine zipper dimer interface shows the proximity of A375C residues on opposed helices, which could allow for the formation of a disulfide bond between monomers. *C*, trypanosome survival (mean ± SD) is plotted after 24 h incubation with the indicated concentrations of APOL1 mutant proteins and compared with APOL1 G0. *D* and *E*, the indicated APOL1 mutant proteins were incubated in chamber buffer under the following pH conditions: (1) 2 h at pH 7.2; (2) 1 h at pH 5.6, followed by 1 h at pH 7.2; or (3) 2 h at pH 5.6. The reactions were then separated by nonreducing SDS-PAGE alongside an equivalent concentration of an untreated control protein (c), and the gel was silver stained. Note that, in addition to the APOL1 monomer band (~43 kDa, m), an APOL1 A375C ~90-kDa dimer band (d) formed specifically when the protein was exposed to first acid then neutral conditions.

As APOL1 traverses the trypanosome endolysosomal pathway, the pH conditions it encounters change from acidic in the endosome to neutral once recycled back to the plasma membrane (Fig. S2). We wanted to understand where in this pathway the ZIP domains may form a coiled coil and facilitate dimer formation of APOL1. The recombinant proteins we utilized contained mutations located at different points along the hydrophobic face of the ZIP domain or proximal to it, in what is predicted to be a turn region between the pore-lining region and the ZIP domain (Fig. 5*A*). APOL1 A363C contains an alanine to cysteine mutation in the turn region, and APOL1 A375C contains an alanine to cysteine mutation in the *a* position of the ZIP domain (Fig. 5*A*). Finally, APOL1 LZA A375C has every *d* position leucine in the ZIP domain substituted for alanines (Fig. 2*A*), in addition to an *a* position alanine to cysteine substitution as in APOL1 A375C (Fig. 5*A*). Figure 5*B* illustrates a space filling model of two interacting heptad repeats containing the A375C mutation, showing proximity of the *a* position A375C cysteines on opposed helices. APOL1 A363C, APOL1 A375C, and APOL1 LZA A375C were first tested in trypanolytic assays (Fig. 5*C*). APOL1 A363C is fully trypanolytic at 800 ng/ml (19 nM) but loses 50% of its activity compared with APOL1 G0 at lower concentrations. The trypanolytic activity of APOL1 A375C is equivalent to APOL1 G0. APOL1 LZA A375C is not trypanolytic at any concentration (Fig. 5*C*).

To biochemically observe dimer formation, we rationalized that if the cysteines in each monomer are close enough to form a disulfide bond, the dimer would be covalently stabilized such that it could be visualized on a nonreducing SDS-PAGE gel, whereas the cysteine-free ZIP domain interaction in APOL1 G0 would be disrupted in nonreducing SDS-PAGE conditions. The pH conditions were selected to mimic the pH changes during trafficking within a trypanosome.

All control APOL1 proteins were thawed on ice fresh from storage at −80 °C and immediately diluted into SDS sample buffer. Test APOL1 proteins were incubated at room temperature at pH 7.2 or pH 5.6. APOL1 A375C did not show disulfide formation after 2 h at neutral pH, suggesting that the cysteines (and thus the ZIP domains) were not close enough to form disulfide bonds under these conditions. Acidification for 1 h followed by neutral conditions for 1 h (to promote disulfide linkage) resulted in an ∼90-kDa disulfide dimer band on a silver-stained, nonreducing SDS-PAGE gel (Fig. 5*D*). Incubation in acidic conditions for 2 h, with only brief neutralization (adjusted to pH 7.2 immediately before addition of sample buffer), showed no disulfide formation.

However, a minority of both APOL1 LZA A375C and APOL1 A363C formed disulfide bands in all three pH conditions tested (Fig. 5, *D* and *E*). The formation of dimer bands by the nontrypanolytic APOL1 LZA A375C occurred during purification and can be seen in the starting material (Fig. 5*E*, lane c). The formation of dimer bands by APOL1 A363C is likely due to the location of the cysteine within the more flexible turn region. This may allow for disulfide trapping in an inactive dimer conformation, as reflected in the partial loss of trypanolytic activity associated with the A363C substitution (Fig. 5*C*). These data indicate that functional dimerization (*i.e.*, ability to lyse trypanosomes) may occur independently of membrane interaction and depends on correct ZIP domain interaction and exposure to acidic pH followed by neutral pH.

### *In silico* analysis of APOL1 channel topology

Having examined the importance of the C-terminal leucine zipper domain for APOL1 trypanolytic activity and open ion channel function, we next sought to identify both the orientation and number of transmembrane domains of the functional APOL1 open-channel state. Currently, there is no consensus pertaining to the number of times the full length APOL1 protein spans the membrane, although earlier reports predicted anywhere between 3 and 5 transmembrane segments (16, 40, 41). We therefore performed *in silico* analysis with the amino acid sequence of APOL1 G0 (GenBank: AAI12944.2) without the signal peptide (residues 28–398). Altogether, the programs identified a combination of five unique putative transmembrane domains (PTM 0–4), with a majority predicting putative transmembrane 1 (PTM 1; 174–198), PTM 2 (205–230), and PTM 4 (335–356) (Table S1, black text). Our data show that APOL1 inserts into bilayer membranes at acidic pH, so we substituted negatively charged aspartate and glutamate residues for isosteric but uncharged and protonated asparagine and glutamine residues before rerunning the membrane prediction software (Table S1, red text). This resulted in TMPred predicting five putative transmembranes (PTM 0–PTM 4), as opposed to only three in the original analysis (PTM 1, 2, and 4).

### Cysteine-scanning mutagenesis coupled with cysteine modification identifies four putative transmembrane regions in the functional open APOL1 cation channel

**Figure 6:**
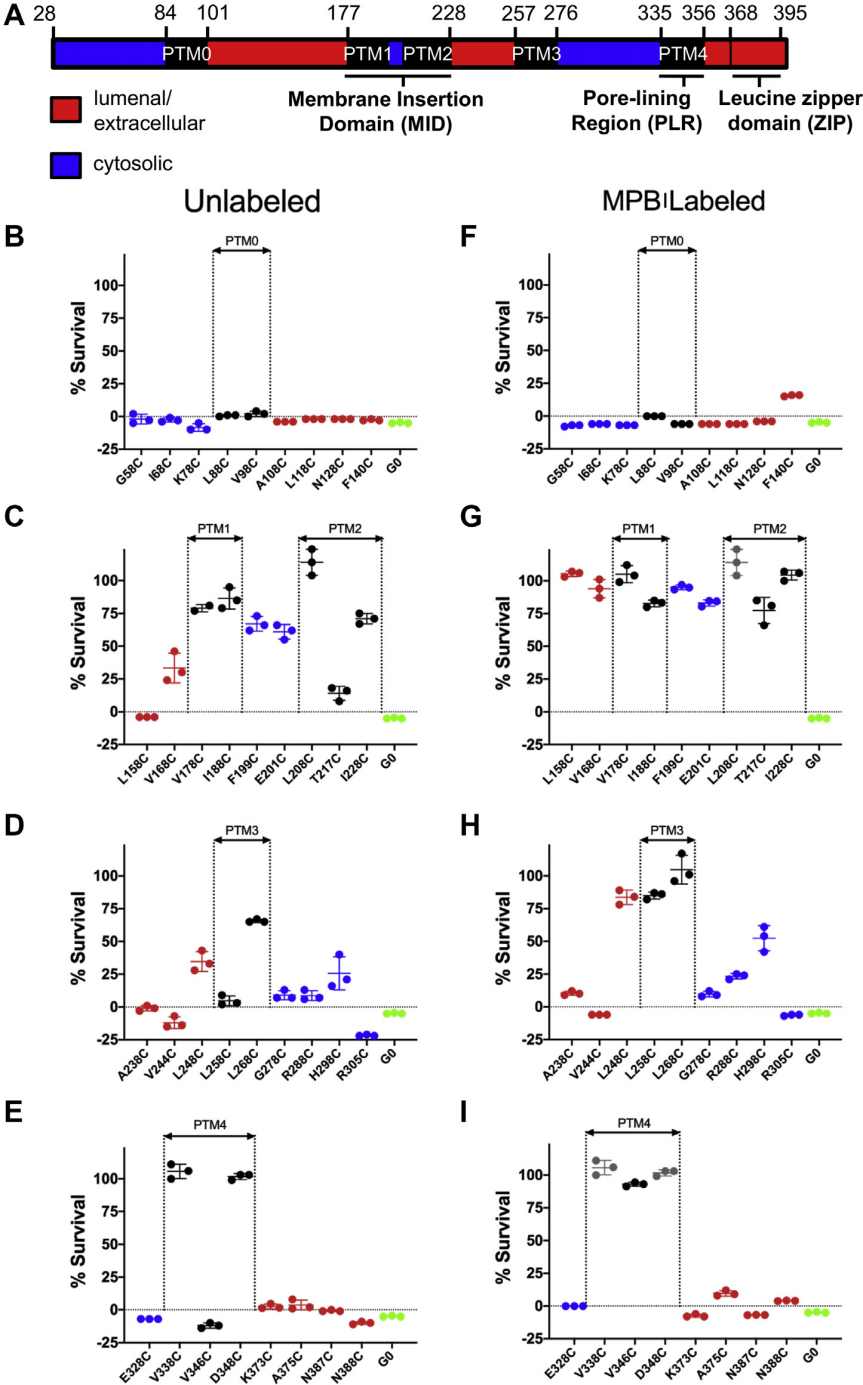
**Trypanolytic activity of cysteine-substituted and MPB-conjugated APOL1.***A*, possible APOL1 topology based on *in silico* prediction. Lumenal/extracellular (*red*), cytoplasmic (*blue*), and putative transmembrane (PTM, *black*). Trypanosome lysis assays of 35 APOL1 cysteine-substituted proteins (*B*–*E*), or the same cysteine-substituted proteins following conjugation with MPB and purification using monomeric-avidin (*F*–*I*). APOL1 proteins with cysteine substitutions in the N terminus (*B* and *F*), MID (*C* and *G*), PTM 3 (*D* and *H*), and PTM 4/PLR plus ZIP (*E* and *I*) regions were assayed. Plotted is mean trypanosome survival ±SD of triplicate measurements, after incubation with the 800 ng/ml of the indicated protein. The *symbol color* indicates the tentative location of the substituted cysteine (extracellular, transmembrane, or cytoplasmic) as indicated in (*A*). The average lytic activity of G0 (N = 10) is shown for reference (*green*). Cysteine-substituted proteins that were inactive in the absence of MPB conjugation were not tested after conjugation. In this case, trypanosome numbers in the absence of MPB conjugation are repeated in the *right-hand panel* for clarity (*grey symbols*). MPB, *N*-(3-maleimidylpropionyl)biocytin.

To probe the five putative transmembrane domains of APOL1, we used protein-wide cysteine-scanning mutagenesis, as APOL1 does not contain endogenous cysteine residues (Fig. 6*A*). Single cysteine substitutions (35 in total) were made at defined positions throughout the polypeptide chain, and the purified proteins were assayed for trypanolytic activity before and after irreversible covalent modification (maleimide bond to the sulfhydryl) with *N*-(3-maleimidylpropionyl)biocytin (MPB; 523.603 Da) (Fig. S3 and Fig. 6, *B*–*I*). We reasoned that APOL1 residues that must insert into membranes to form functional open APOL1 cation channels might be identified upon modification with the large, hydrophilic MPB moiety, which would prevent their insertion into membranes and result in inhibition of trypanolytic activity.

To begin elucidating the topology of the functional open APOL1 cation channel, we tested each cysteine-substituted APOL1 protein for trypanolytic activity against *T. b. brucei in vitro*. Cysteine substitutions within the N-terminal region (between residues 28 and 140) were well tolerated, whether MPB modified or not, with no observable loss of trypanolytic function (Fig. 6, *B* and *F*). In contrast, all but one of the unmodified cysteine substitutions generated within the MID, encompassing putative transmembrane regions PTM 1 and PTM 2 (residues 177–228), were detrimental to activity (Fig. 6*C*). Modification with MPB exacerbated this effect, causing inhibition of trypanolytic activity when coupled to all MID residues tested (Fig. 6*G*). The deleterious effect of MPB modification also affected residues located within 19 amino acids N-terminal of the MID (APOL1 L158C-MPB and APOL1 V168C-MPB). These are within the newly designated helix 5 in the N-terminal domain and likely interfere with inter-protein folding (34). Overall these data confirm that the MID and helix 5 are critical for trypanolytic activity.

**Figure 7:**
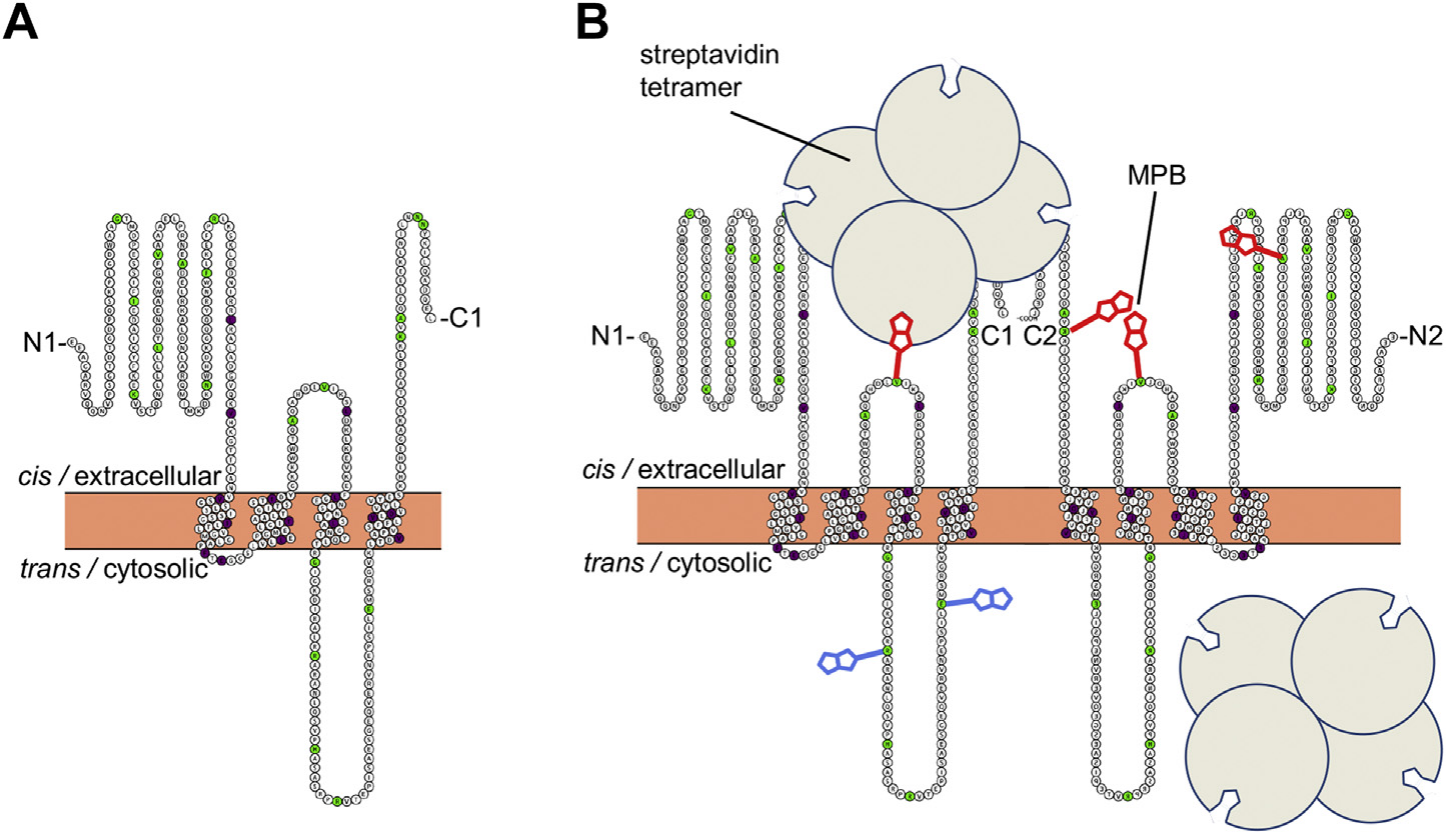
**Working model of APOL1 G0 channel topology and its interrogation by substituted cysteine accessibility method.***A*, Protter diagram (59) of APOL1 topology incorporating *in silico* (Table S1) and MPB modification results (Fig. 6). Note that the structure of the N terminus has been recently solved (34) whereas loop structures are unknown. *Filled circles* indicate Cys substitutions or Cys-MPB modifications that increased trypanosome survival to greater than 50% (*purple*), or had no effect on trypanosome lysis (*green*) when incubated with trypanosomes at 800 ng/ml for 24 h. Note that PTM 0 was excluded owing to both a marginal *in silico* prediction and a lack of functional inhibition when MBP was conjugated to residues in this region. *B*, to test the 4-TM model of the APOL1 channel, the *cis versus trans* accessibility of an MPB-conjugated residue can be probed by SCAM. First, a purified (and trypanolytic) MPB-conjugated APOL1 protein is allowed to form pH-dependent channels in planar lipid bilayers under standard conditions. Streptavidin (a 53-kDa tetramer) is then added to either the *cis* or the *trans* side. If the streptavidin can bind to the Cys-MPB residue it may affect some measurable property of channel function (*e.g.*, channel conductance or pH gating), owing to steric effects on the channel’s three-dimensional structure. The MPB-conjugated residue is thus mapped to either the *cis* or the *trans* side of the channel based on its accessibility to *cis versus trans* streptavidin. Alternatively, if streptavidin addition to both sides has no effect on channel properties (either because MPB is not accessible or because binding to MPB has no measurable effect) no conclusion can be made regarding the position of Cys-MPB. In the hypothetical example shown, *red* MPB is conjugated to a cysteine residue that is accessible to streptavidin binding on the *cis*, but not the *trans*, side. Some of the additional positions that were tested on the hypothetical *trans* side, *blue* MPB are also shown. MPB, *N*-(3-maleimidylpropionyl)biocytin.

PTM 3, located C-terminal of the MID, was predicted to span residues 257 to 276 (Table S1, JPRED-4 and TMPRED). Of interest, the APOL1 L268C substitution reduced trypanolytic activity even without MPB labeling (Fig. 6*D*). In addition, MPB modification of APOL1 L258C, as well as PTM 3 flanking residues L248C-MPB and H298C-MPB, was associated with a reduction of trypanolytic activity (Fig. 6*H*). These results provide additional support for the existence of a putative transmembrane region (PTM 3) between the MID (PTM 1 and 2) and the pore-lining region (PTM 4) of the functional open APOL1 cation channel. Within the final putative transmembrane domain (PTM 4), which is also the pore-lining region, three different cysteine substitutions resulted in complete loss of trypanolytic activity, either on their own (V338C and D348C; Fig. 6*E*) or after coupling to MPB (V346C-MPB; Fig. 6*I*). These results support a four-transmembrane model of the functional open APOL1 cation channel (Fig. 7*A*).

### Substituted cysteine accessibility method determines the orientation of the open functional APOL1 cation channel in planar lipid bilayers

**Figure 8:**
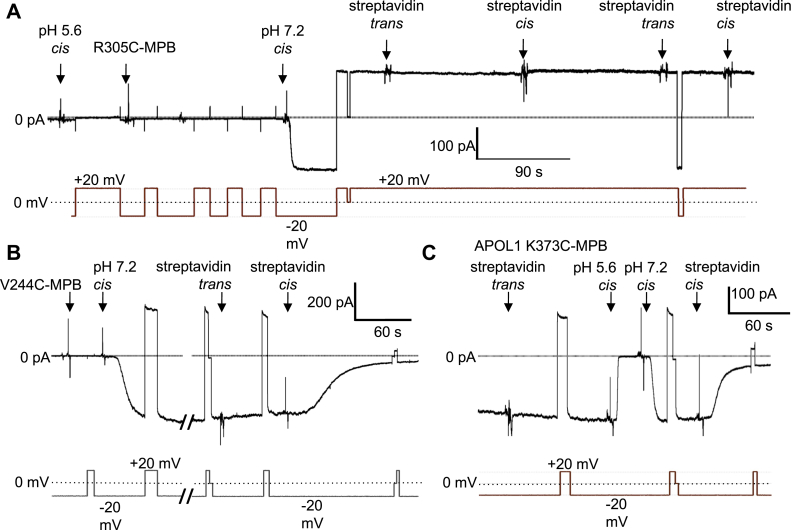
**Representative effects of streptavidin addition on the conductance formed by APOL1 Cys-MPB proteins in planar lipid bilayers.** In each case the voltage was set by the experimenter (*lower trace*) as the current was recorded (*upper trace*). *A*, starting with symmetric pH 7.2 conditions the *cis* side was first adjusted to pH 5.6 and then 9.3 nM APOL1 R305C-MPB was added. After allowing for bilayer insertion, the *cis* side was adjusted to pH 7.2 resulting in the usual conductance increase. Streptavidin (94 nM) was added to first the *trans* and then the *cis* sides. In neither case was there an appreciable effect on the conductance, meaning no conclusion can be made about the position of R305C-MPB. *B*, with *cis* pH 5.6, *trans* pH 7.2 conditions 9.3 nM APOL1 V244C-MPB was added and then allowed to insert into the bilayer. The *cis* chamber was then adjusted to pH 7.2 with KOH, resulting in the expected conductance increase. During the break in the record the *cis* side was perfused with chamber buffer (pH 7.2) to remove free biotinylated protein. Streptavidin (94 nM) was added first to the *trans* side, which had no effect on the conductance, and then to the *cis* side, which caused a substantial decrease in conductance, indicating that V244C-MPB is accessible to *cis* but not *trans* streptavidin. *C*, before the start of the record, a conductance was obtained with 9.3 nM APOL1 K373C-MPB in the usual way, and *cis* side was perfused with chamber buffer (pH 7.2). Streptavidin (94 nM) was then added to the *trans* side as indicated, which had no effect on conductance. The *cis* side was then acidified (pH 5.6) and neutralized (pH 7.2) to demonstrate normal pH gating. After *cis* addition of 94 nM streptavidin there was a significant decrease in conductance, indicating that the modified leucine zipper domain K373C-MPB is accessible to *cis*, but not *trans*, streptavidin. In all cases the data shown are representative of three independent experiments. MPB, *N*-(3-maleimidylpropionyl)biocytin.

Having identified MPB-labeled residues that inhibited trypanolytic function, we next sought to interrogate APOL1 channel topology by focusing on the APOL1 Cys-MPB proteins that retained trypanolytic function, marked as green circles in the Protter diagram of APOL1 (Fig. 7*A*). After the formation of pH-gated channels by the MPB-modified proteins in planar lipid bilayers, we added streptavidin to either side of the bilayer (42, 43). Streptavidin is a 53-kDa tetramer that may sterically interfere with APOL1 channel structure/function if it can bind to the MPB-conjugated residue, resulting in a measurable change in some property of channel activity (*e.g.*, ion flow or pH gating). This technique allowed us to map an MPB-conjugated residue to either the *cis* or *trans* side of the planar lipid bilayer membrane if streptavidin addition resulted in a site-specific change to the macroscopic conductance (Fig. 7*B*). By these criteria we identified nine residues that could not be mapped because streptavidin addition to either the *cis* or *trans* side had no effect on the conductance or pH gating (Table S2 and Fig. 8*A*).

**Figure 9:**
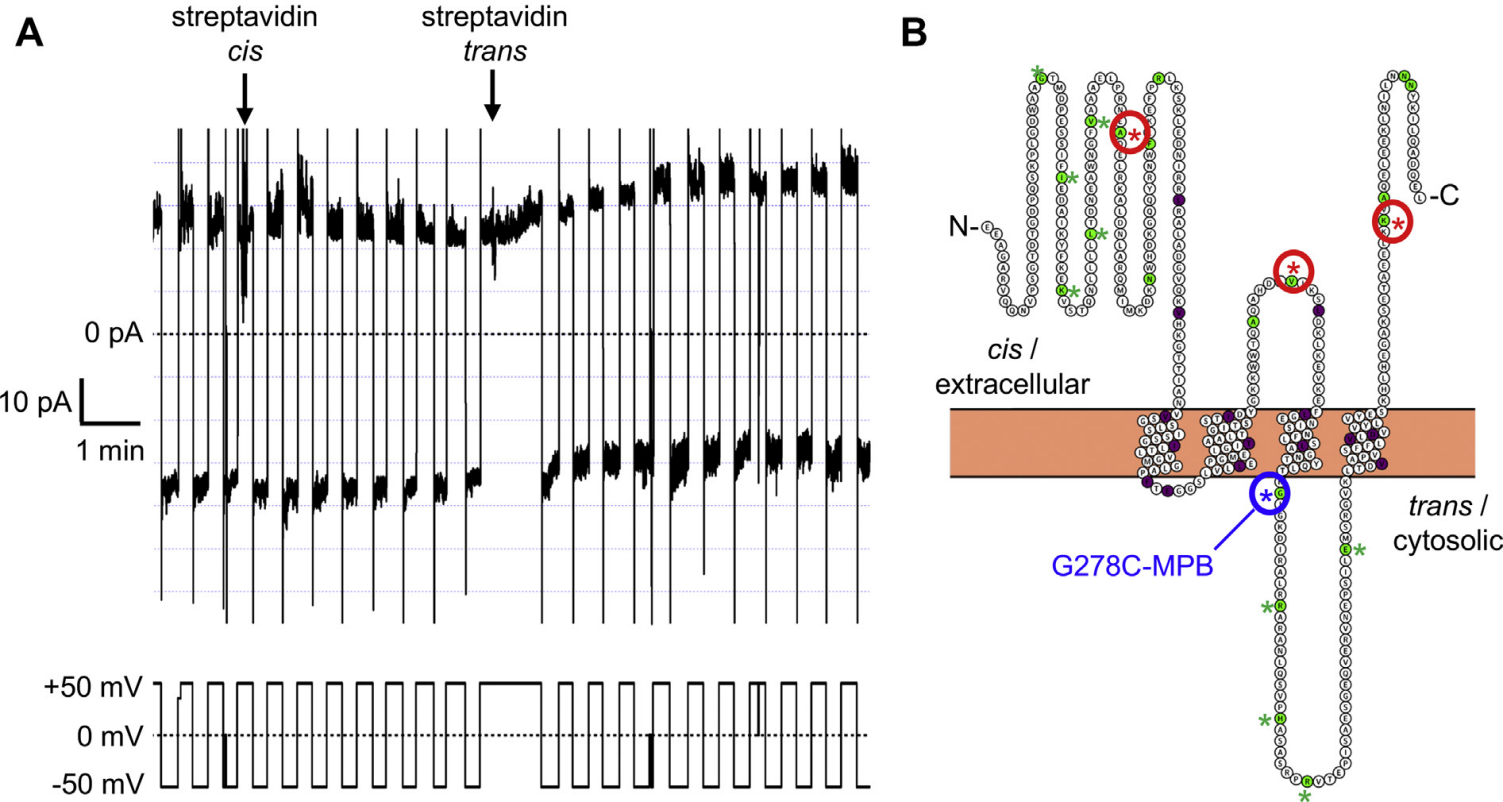
**The conductance formed by APOL1 G278C-MPB is affected by *trans* streptavidin.***A*, before the start of the record, APOL1 G278C-MPB (6 nM) was added to the membrane (*cis* pH 5.6, *trans* pH 7.5) until a detectable conductance was obtained. The *cis* pH was then adjusted to pH 7.5 to allow for channel opening. The voltage was alternated between ±50 mV (*lower trace*) and the current was recorded as shown (*upper trace*). As is typical of APOL1, the current magnitude was greater at negative voltage than at positive voltage. This remained true after addition of streptavidin (94 nM) to the *cis* side as indicated. However, after streptavidin (94 nM) was added to the *trans* side, the voltage effect on the current was reversed: the current magnitude became greater at positive than at negative voltage. The data shown are representative of three independent experiments. *B*, Protter diagram showing proposed membrane topology of an APOL1 monomer subunit in the active open cation channel state. The structure of the N terminus has been recently solved (34) whereas loop structures are unknown. *Filled circles* indicate the position of Cys-MPB modifications that yielded “functional” (<50% trypanosome survival, *green*), or “nonfunctional” (>50% trypanosome survival, *purple*) APOL1 proteins when incubated with trypanosomes at 800 ng/ml for 24 h. *Asterisks* mark the positions of MPB-modified cysteines that were tested for accessibility to both *cis* and *trans* streptavidin. The *blue asterisk* marks G278C-MPB, which affected function when incubated with *trans* streptavidin; the *red asterisks* mark residues A108C-MPB, V244C-MPB, and K373C-MPB, which affected function when incubated with *cis* streptavidin, and the *green asterisks* mark residues that did not affect function when incubated with either *cis* or *trans* streptavidin. *Green residues without asterisks* were not tested with streptavidin. MPB, *N*-(3-maleimidylpropionyl)biocytin.

**Figure 10:**
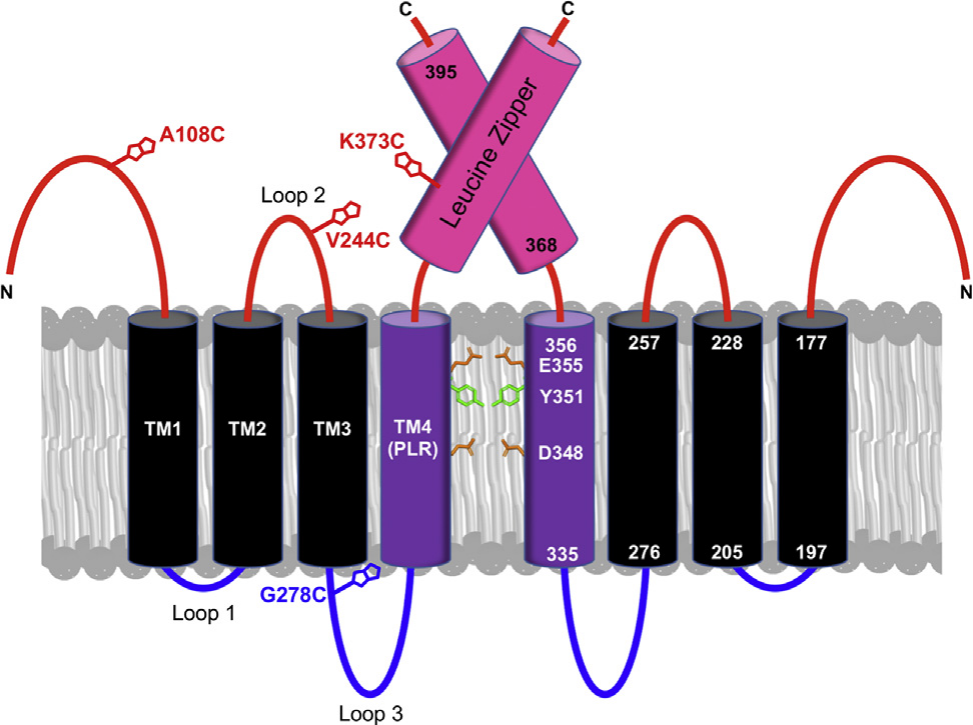
**Model of the open APOL1 cation channel.** The channel is shown as a dimer wherein the pore-lining region of the two monomers are brought together by a coiled-coil interdigitation between the C-terminal leucine zipper domains. The leucine zipper could also allow for the formation of trimer or tetramer channels (see Discussion section). Each subunit contributes four transmembrane domains to the channel structure, with TM 1 and TM 2 constituting the membrane insertion domain and TM 4 constituting the pore-lining region (PLR). The positions of Cys-MPB residues that were mapped by substituted cysteine accessibility method analysis are indicated: A108C, V244C, G278C, and K373C. Also shown are the pore-lining residues responsible for pH gating and cation selectivity: D348, Y351, and E355 (16). Structure of the N terminus has been recently solved (34) whereas loop structures are unknown.

Significantly, in the case of three different MPB-conjugated APOL1 proteins, streptavidin addition to the *trans* side had no effect, but the subsequent addition of streptavidin to the *cis* side caused a decrease in the macroscopic conductance (Table S2 and Fig. 8, *B* and *C*). These three MPB-conjugated residues (A108C-MPB, V224C-MPB, and K373C-MPB) were therefore mapped to the *cis* side of the open cation channel state. Finally, the conductance induced by APOL1 G278C-MPB was unaffected by *cis* streptavidin, but was affected by the subsequent addition of *trans* streptavidin, causing a clear change in the voltage dependence of the macroscopic conductance (Fig. 9*A*). Neither *cis* nor *trans* addition of streptavidin to unconjugated APOL1 G0 had any such effect on the conductance (Fig. S4). These data are consistent with A108C-MPB and V244C-MPB being positioned on opposite ends of the MID (PTM 1 and 2), and with G278C-MPB being positioned at the *trans* end of a third transmembrane domain (PTM3), which must occur between V244 and G278 (Fig. 9*B*). The PLR would then form the fourth and final transmembrane domain (PTM 4), with the C-terminal leucine zipper domain (which contains K373C-MPB) being positioned on the *cis* side of the channel (Figs. 9*B* and 10). These results effectively rule out a two-pass transmembrane model (PTM 1 and 4; Table S1, HMMTOP), in which V244C-MPB would be positioned on the *trans* side. These results also rule out the three-pass model favored by the secondary structure prediction (Table S1), in which K373C would be present on the *trans* side. Overall, our data strongly support a four-transmembrane model of the open cation channel state (summarized in Fig. 9*B*). The final proposed model of the dimerized open and functional APOL1 cation channel is presented in Figure 10.

## Discussion

In this article, we have addressed the oligomeric state and membrane topology of the functional APOL1 channel. Using site-directed mutagenesis and chemical modification, we show that a C-terminal heptad-repeat leucine motif constitutes a *bona fide* ZIP domain that mediates coiled-coil (knobs-into-holes) interdigitation facilitating dimerization during APOL1 channel formation. Then, using cysteine-scanning mutagenesis and the substituted-cysteine accessibility method (SCAM), we provide evidence that the APOL1 channel has four transmembranes per monomer in the open ion channel conformation and show that both the N- and C-terminal regions of the protein are on the *cis* (lumenal/extracellular) side of the lipid bilayer membrane. Finally, we present an oligomeric topology model of the APOL1 channel, in which both channel lining and oligomerization functions are contributed by separate subdomains of the C-terminal domain.

Contrary to initial reports (5, 33), we and others have shown that the C-terminal domain (aa 335–398) is essential for the formation of APOL1 cation channels and trypanolytic function (6, 14, 35). In a previous report we showed that residues D348, Y351, and E355 form the pore-lining region and are critical to the pH gating and/or cation selectivity functions of the APOL1 channel (16). In this paper, we focused on the C-terminal leucine zipper domain as a possible mediator of APOL1 protein–protein interactions. When we substituted the four leucines (371A/378A/385A/392A) occupying the heptad repeat *d* positions to alanine (APOL1 LZA; Fig. 2*A*), the resulting protein was unable to either lyse *T. b. brucei in vitro* (Fig. 2*B*) or form stable conductances (ion flow) in planar lipid bilayers, despite evidence of membrane insertion at *cis* pH 5.6/*trans* pH 7.2 (Fig. 3*C*). Moreover, sequential substitutions of the leucines, from either the N-terminus or the C-terminus of the ZIP domain (LZA 1–6; Fig. 2*A*), highlighted codependence of the leucines in channel formation, as the number of alanine substitutions correlated with loss of trypanolytic activity (Fig. 2, *C* and *D*). The individual leucine substitution at the first heptad position (L371A) had a significant impact on trypanosome lysis, whereas the substitution at the final heptad position (L392A) did not. This confirms an earlier observation that the African variant APOL1 G2, which also lacks a leucine in the fourth heptad position due to a two amino acid deletion (N388/Y389), was fully trypanolytic *in vitro* (14). Another group found that a nine-amino-acid deletion from the C-terminus, which also removed the L392 residue, retained lytic activity (35). Thus, while the entirety of the heptad repeat motif is not required, our data suggest that heptad-repeat leucines provide a hydrophobic interface that interdigitates with a second APOL1 protein to promote coiled-coil oligomeric channel formation.

To further test this model, we introduced cysteines into either the hydrophobic face (A375C) or the hydrophilic face (K373C) of the N terminus of the ZIP domain amphipathic helix (Fig. 4, *A* and *B*) and asked whether preincubation with MTSET (278.2 Da) could sterically hinder channel formation in planar lipid bilayers (Fig. 4, *C* and *D*). APOL1 A375C-MTSET was unable to form an open cation channel upon *cis* pH neutralization (Fig. 4*D*), whereas APOL1 K373C-MTSET generated channels similarly to unlabeled APOL1 (Fig. 4*E*). These data indicate that interfering with the hydrophobic face of the amphipathic helix inhibits the formation of functional channels. Moreover, APOL1 A375C formed disulfide-linked dimers after acidification and subsequent neutralization, as would occur during APOL1 channel formation (Fig. 5*D*). We conclude that the APOL1 C-terminal domain contains a ZIP domain, which mediates oligomeric APOL1 channel formation *via* coiled-coil interdigitation between leucine zipper helices. Thus, the C-terminal domain (aa 335–398) contains both the pore-lining region and an oligomerization domain, which are both required for the formation of functionally active APOL1 cation channels. These data further highlight the central role of the C-terminal domain in channel formation.

Based on the evidence presented that a coiled-coil interaction is required for APOL1 open cation channel activity, we will speculate here on the oligomeric state of the APOL1 cation channel. Naturally occurring proteins with heptad repeats (*e.g.*, leucine zippers) are able to form multiunit coiled coils ranging from dimers to hexamers (44). However, it has been demonstrated that the identity of the ZIP domain heptad-repeat *a* and *d* positions strongly predicts the oligomeric state of the leucine zipper-type coiled coil (38). Utilizing the ZIP domain of the yeast transcription factor GCN4, this group revealed switching of the *a* and *d* positions between isoleucine–leucine, isoleucine–isoleucine, and leucine–isoleucine exclusively formed dimers, trimers, and tetramers, respectively. In the case of APOL1, only leucines are observed in the *d* position of the heptad repeat, which might suggest that the APOL1 ZIP domain is either a dimer or trimer, similar to the leucine zipper domain of GCN-4 (45). However, differing residue identities at the APOL1 *a* positions compared with those of the GCN-4 make the potential oligomeric status of the APOL1 leucine zipper difficult to predict.

With regards to the pH dependence of channel formation, we speculate that acidification allows for membrane insertion of the MID (residues 176–228) due to protonation and charge neutralization of MID glutamate residues E201, E209, and E213. Concurrently, charge neutralization of the C-terminal ZIP domain (368–395; pI of ∼4.4) at acidic pH would promote the leucine zipper coiled-coil interaction and oligomeric channel formation. This idea is further supported by the oligomerization experiments, in which preincubation at pH 5.6 was necessary for the spontaneous formation of disulfide-linked homodimers by the APOL1 A375C protein (Fig. 5). Accordingly, APOL1 LZA and MTSET-conjugated APOL1 A375C could insert into lipid bilayers to form a minor conductance at pH 5.6 (membrane inserted “closed” channel), but neither protein could form open channels upon *cis* pH neutralization, indicating that preoligomerization is not necessary for membrane insertion (Figs. 3*C* and 4*D*). Moreover, the ability of APOL1 A375C to form covalent dimers in the absence of membranes supports the idea that dimerization and membrane insertion of the MID are independent processes that both occur at acidic pH (Fig. 5*D*) (16).

The results presented here are also relevant to the mechanism by which the SRA of *Trypanosoma brucei rhodesiense* blocks APOL1-mediated lysis. A past study showed that SRA interacts with a C-terminal peptide of APOL1 under acidic conditions and that a single amino acid substitution within the leucine zipper domain (N388K) reduced this interaction (40). By binding to the ZIP domain, SRA may block oligomerization of APOL1 and prevent the formation of the cation channels that cause trypanosome swelling and lysis. Our experiments show that the presence of SRA protein limited the formation of disulfide-linked APOL1 A375C homodimers, after preincubation at acidic pH (Fig. S5). Our results are also in agreement with a previous report that preincubation of APOL1 with SRA at acidic pH did not prevent APOL1 membrane insertion, rather the interaction with SRA prevented the formation of pH-gated cation channels and allowed APOL1 to be removed from the membrane upon subsequent pH neutralization (14). Taken together, the above evidence strongly suggests that the APOL1 channel is an oligomer that forms from the association of membrane-bound monomers (prechannel form) and that SRA prevents oligomer formation by interacting with the ZIP domain.

Humans with two copies of APOL1 renal risk variants G1 (dbSNP: rs73885319 and rs60910145) and G2 (rs71785313) have a higher risk of developing kidney disease than those with the APOL1 G0 allele (GenBank: EAW60091.1 and AAI12944.2). This risk is associated with increased cytotoxicity of the APOL1 renal risk variants when expressed in human cells or in mouse kidneys, although overexpression of APOL1 G0 is also cytotoxic. Currently, several mechanisms have been invoked to account for cytotoxicity caused by APOL1 renal risk variants, including but not limited to formation of channels at the plasma membrane leading to calcium and sodium influx/potassium efflux (29, 30), opening of the mitochondrial permeability transition pore (46), interaction with APOL3 (47), differential localization to lipid droplets (48), activation of the inflammasome (49), and/or differences in potassium flux between variants (50). We found that the ZIP domain mutant APOL1 LZA was less cytotoxic than APOL1 G0 (GenBank: AAI12944.2) when overexpressed in human cells (Fig. S1). Although both the G1 (S342G, I384M; rs73885319 and rs60910145) and G2 (N388, Y389 deletion; rs71785313) amino acid substitutions are within or proximal to the ZIP domain, none of them are expected to alter oligomerization of the ZIP domain directly. S342G is in the PLR, whereas the two changes within the ZIP domain are either relatively conservative (I384M) or, in the case of G2, disruptive to the fourth turn of ZIP helix, which appears to have little impact on trypanolytic activity (Fig. 2 and see Discussion above). Indeed, the G1 and G2 substitutions were previously found to alter the stability of alternative (*i.e.*, non-channel forming) conformations of the C-terminal domain (51, 52). These authors predicted and measured by NMR the presence of a hairpin structure formed by the ZIP domain and PLR. We hypothesize that this hairpin maintains the ion channel in an inactive form. It was further demonstrated that the G1 and G2 hairpins were less stable than the G0 hairpin (51). We hypothesize that this instability allows for enhanced formation of the ZIP dimer and release of the PLR, for insertion into the membrane, which results in active open cation channels. Therefore, the upstream activator of APOL1-mediated kidney disease may be increased dimerization and channel formation by G1 and G2 owing to decreased hairpin stability of the monomer. This possibility will require further investigation in the future.

In this paper, we also addressed the membrane topology of the APOL1 channel. An *in silico* approach was utilized to identify potential transmembrane models that we could begin to test *in vitro* (Table S1). Altogether, a total of five putative transmembrane domains (PTM 0–4) were identified, although only three were identified by most prediction servers, namely, the two transmembrane domains of the MID (residues 177–228, PTM 1 and 2) and the pore-lining region between residues 335 and 356 (PTM 4), which we identified previously (16). However, additional PTMs were identified by individual prediction servers between residues 84 and 101 (PTM 0) and between residues 257 and 276 (PTM 3).

To test the *in silico* predictions, we used cysteine-scanning mutagenesis coupled with MPB modification to identify functionally important residues in the APOL1 channel, reasoning that residues in transmembrane domains would be negatively affected by modification. Indeed, such residues were found to cluster in or around PTMs 1 to 4, but we found no evidence to support a role for PTM 0 (Figs. 6 and 7*A*). Of importance, this four-transmembrane model (PTM 1–4) is consistent with appropriate *cis* (extracellular/lumenal) positioning of the *cis* pH-sensing residue glutamate 355 (16). Moreover, our SCAM analysis allowed us to identify three modified residues (A108C-MPB; V244C-MPB, and K373C-MPB) that reacted with *cis*-added streptavidin, causing a decrease in the APOL1-induced conductance (ion flow) under conditions that favor the open channel state (Fig. 8 and Table S2). Also under these conditions, we found that G278C-MPB was accessible to *trans*-added streptavidin, in accordance with the proposed position of G278 at the *trans* end of PTM 3 (Fig. 9 and Table S2). Taken together, these data strongly support a four-transmembrane model for the active open APOL1 cation channel, with the PLR forming the fourth and final transmembrane domain (Figs. 9*B* and 10). This model is reminiscent in certain respects to the human voltage-gated proton channel, which notably has four transmembrane domains, a C-terminal heptad repeat allowing for channel dimerization, and an aspartate residue that determines selectivity for protons (53, 54).

Recently, the topology of plasma membrane–associated APOL1 was determined by epitope mapping after expression in immortalized podocytes (32). In common with our analysis, epitopes N-terminal of PTM 1/2 and within the C-terminal ZIP domain (residues 368–395) were accessible to monoclonal antibodies on the extracellular (*cis*) side of the plasma membrane. However, the region directly N-terminal of PTM 4 (PLR) was also exposed on the extracellular (*cis*) side, leading the authors to conclude that no transmembrane region exists between residues 257 and 276 (PTM 3) and that PTM 4 is more likely an intramembrane loop rather than an actual transmembrane domain. Clearly there are many methodological differences between the two studies that could explain this disparity. In the epitope mapping study, APOL1 was produced in the endoplasmic reticulum of a human cell and then the entire population of APOL1 protein was probed at the surface of the same cell. In the current study, recombinant APOL1 was purified from *Escherichia coli* and then the open channel conformation was probed after incorporation into artificial planar lipid bilayers, in the absence of any cellular cofactors. However, after association with planar lipid bilayers at neutral pH, recombinant APOL1 exists in a membrane bound, preactive conformation, which can be slowly converted into an active cation channel at acidic *cis* pH 6.2 (Fig. S6). Since epitope mapping cannot distinguish between conformational substates, we hypothesize that the majority of plasma membrane–associated APOL1 also exists in a preactive, nonchannel conformation and that this resembles the state detected by Gupta *et al.* (32). According to our model, active open cation channel formation would therefore require full transmembrane insertion of PTM 3 and PTM 4 at acidic pH and the concomitant translocation of the intervening region (residues 276–305) from the *cis* to the *trans* side of the bilayer. This is in accordance with the placement of G278C-MPB on the *trans* side of the open channel state (Figs. 9 and 10) and the susceptibility of the cation channel conductance to the *trans* addition of proteinase-K (Fig. S7).

Taken together, the evidence presented in this paper suggests that, under acidic conditions, APOL1 can insert into planar lipid bilayers and oligomerize *via* a C-terminal ZIP domain to form a functionally active, pH-gated cation channel (Fig. 10). We show that each APOL1 monomer likely contributes four transmembrane domains to the functionally active, open cation channel state, such that both the N- and C-terminal regions of the protein reside on the *cis* side of the bilayer membrane. In this model, both channel-lining and oligomerization functions are contributed by separate subdomains of the C-terminal domain. Our data suggest that the resistance of the human-infective trypanosome *T. b. rhodesiense* to APOL1-induced lysis can be explained by the ability of SRA to inhibit leucine zipper interactions between subunits of the APOL1 channel. Furthermore, the dependency of channel activity on oligomer formation may also have implications for APOL1-mediated kidney disease.

## Experimental procedures

### PCR mutagenesis and transformation of bacteria

Recombinant APOL1 was expressed from the pNIC28 vector (Addgene) containing an N-terminal 6× His-tagged G0 APOL1 coding sequence (aa 28–398). The most globally common isoform was used (K150, I228, K255; GenBank: AAI12944.2) (55). Individual cysteines were substituted into this plasmid using site-directed mutagenesis (QuikChange II, Agilent Technologies). Around 25 ng of plasmid DNA was mixed with 125 ng of forward and reverse primers, dNTPs, 10× PfuUltra reaction buffer, and PfuUltra DNA polymerase (Agilent, 600385). After 18 cycles of PCR were performed, template DNA was digested with Dpn1 (Agilent, 500402) under standard conditions. The PCR product was transformed into chemically competent Stellar cells (Takara, 636763), which were allowed to recover in Special optimal broth with catabolite repression (SOC) medium (Takara, ST0215). The purified plasmid product was sequenced to ensure only the correct mutation was made.

### APOL1 protein purification

Recombinant APOL1 protein was purified as described (16, 56). Briefly, *E. coli* BL21-DE3-RIPL cells (Agilent) were transformed with the purified pNIC28 plasmid, and grown for ∼16 h in overnight express-terrific broth autoexpression media (Novagen, 714915). Bacterial pellets were first resuspended and then sonicated in lysis buffer (50 mM Tris-HCl, pH 8.0, 1 mM EDTA supplemented with 0.5 mM DTT and 0.5 mM PMSF), and then 100 μg/ml lysozyme, 5 μg/ml DNAse1, and 5 μg/ml RNAse1 were added. The suspension was stirred for 30 min at room temperature (RT) and then 0.5% (w/v) sodium deoxycholate (made fresh) and 0.5% Triton X-100 (Surfact-AMPS, Pierce) were added, together with 3.5 mM MgCl_2_ and 2.5 mM CaCl_2_ to stimulate nuclease activity. After an additional 30 min stirring at room temperature, the suspension was sedimented at 26,000*g*, resuspended in 1% Triton X-100 and 100 mM NaCl in lysis buffer, and then stirred for an additional 30 min at room temperature. After further washes with 100 mM NaCl in lysis buffer, followed by 20% (v/v) lysis buffer in water, the pellet was then solubilized in 2% (w/v) zwittergent 3-14 (Calbiochem) and purified by nickel-chelate chromatography (GE, 17524701), followed by size exclusion chromatography (GE, 28990944) as detailed previously. The purified protein, still dissolved in SEC buffer (50 mM Tris-HCl, pH 8.5, 150 mM NaCl, 0.05% dodecyl maltoside), was flash frozen on dry ice in small aliquots (<0.1 ml) and stored at −80 °C. For cysteine APOL1 mutants, the final SEC buffer contained 1 mM DTT to prevent the formation of spontaneous disulfide linkages. The purified proteins were then run on an SDS-PAGE gel (Bio-Rad, 4569034, and Thermo Scientific PageRuler Plus Prestained 10–250 kDa Protein Ladder, PI26620) and Coomassie stained (Coomassie Brilliant Blue R-250, Fisher BP101-25) to confirm correct predicted band size and minimal contamination. In all experiments the proteins were used without removing the N-terminal His tag.

### MTSET conjugation

MTSET (Biotium, 91021) is a sulfhydryl-reactive, membrane-impermeable compound that rapidly forms stable disulfide bonds with substituted cysteines at neutral pH, resulting in the addition of a charged ethyltrimethyl ammonium group (see later discussion). The reagent was conjugated to cysteine-substituted APOL1 as described in the legend to Figure 4. We used a 250,000-fold molar excess of MTSET to overcome limited stability of the compound in aqueous solutions at neutral pH.

**Figure:**
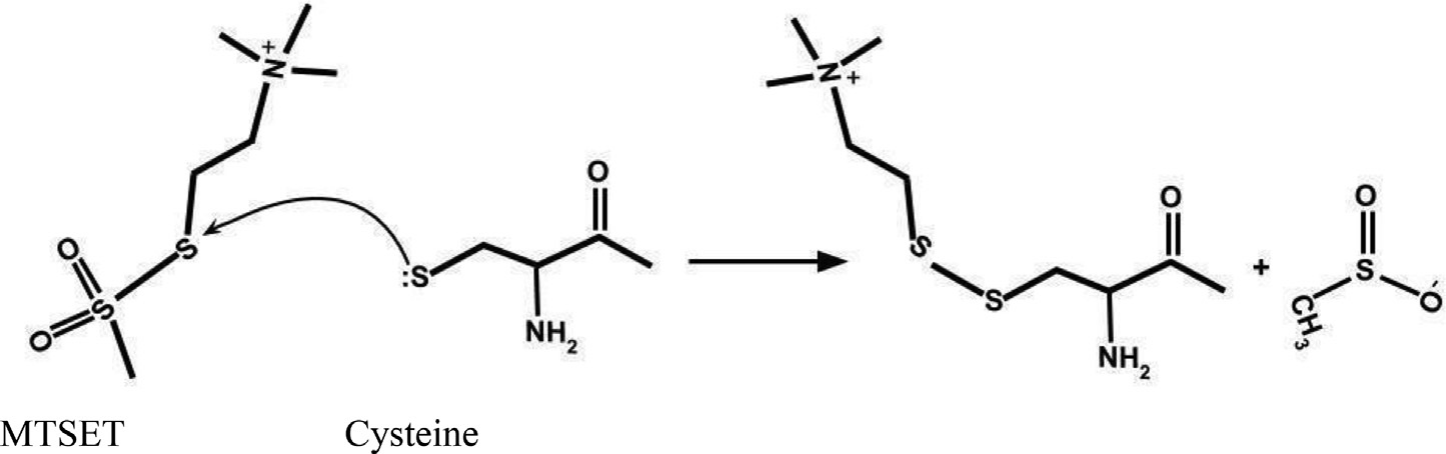

### MPB conjugation and purification of MPB-conjugated proteins

Approximately 0.45 ml of rAPOL1 protein (∼20–100 nM) was incubated with 50 μl of 1 mM MPB (100 μM) for 1 h at room temperature. MPB forms a covalent imide bond with free cysteines, which cannot be broken in reducing conditions. After incubation, proteins were added to a 2-ml Centrifuge Column (Pierce, 89896) packed with 1 ml of monomeric avidin bound to Sepharose beads (Pierce, 20228). The flow-through was discarded, and the bound MPB-labeled APOL1 protein was eluted with SEC buffer (see above) containing 2 mM biotin. The excess biotin was removed by repeated rounds of washing in a centrifugal filter unit with a 10-kDa molecular weight cutoff (Amicon, UFC901024). The resulting protein solution was then concentrated down to 0.5 ml, aliquoted, and flash frozen in liquid nitrogen for storage at −80 °C. To determine MPB conjugation efficiency (Fig. S3) the biotinylated proteins were incubated with streptavidin (Molecular Probes, 434302)

### Trypanosomes and 24-h trypanosome lysis assays

The Lister-427 derived cell line *T. b. brucei* 427 was cultured in HMI-9 media (57). Trypanolytic assays were performed in triplicate, in sterile, opaque 96-well plates. As a positive control for trypanolysis, 0.1 ml of 0.05% n-Dodecyl-B-D-Maltoside was diluted with 0.1 ml HMI-9 for a final concentration of 0.025% n-Dodecyl-B-D-Maltoside; 0.1 ml of HMI-9 culture media was used as a negative control for trypanolysis. Dilutions, 0.1 ml, of the various rAPOL1 mutant proteins were plated in triplicate. *T. b. brucei* 427 were diluted to 5 × 10^5^ cells/ml, and 0.1 ml was added to each well. Trypanosomes were incubated with rAPOL1 for 20 h at 37 °C with 5% CO_2_. Viability was then assessed by incubating with 20 μl of alamarBlue (Invitrogen, DAL1100) in each well for four additional hours at 37 °C, followed by measurement of fluorescence with the Gemini XPS Microplate Reader plate reader (Ex: 530 nm; Em: 590 nm). Data were analyzed and graphed using Prism 7 software by GraphPad.

### Planar lipid bilayers

Planar lipid bilayers were formed in an apparatus of two 1-ml chambers separated by a Teflon partition with a 100-μm hole essentially as described (14, 16). The lipid solution (stored at −20 °C between uses) consisted of 1.5% asolectin, from which nonpolar lipids had been removed (55) and 0.5% cholesterol (w/v) (Sigma) dissolved together in pentane. Before addition of this lipid solution to the chambers, the Teflon partition was treated on each side with 2 to 4 μl of a 3% (v/v) squalene solution (Sigma) dissolved in petroleum ether. A small volume (0.275 ml) of chamber buffer (150 mM KCl, 0.5 mM EDTA, 5 mM CaCl_2_, 5 mM K-succinate, 5 mM K-Hepes, adjusted to pH 7.2 or 5.6 as needed and stored at 4 °C between uses) was applied to the base well of each chamber, with 20 μl of the asolectin/cholesterol solution subsequently added on top. Care was taken to avoid contact of the asolectin solution with the partition. The apparatus was left for several minutes undisturbed to allow for pentane evaporation. Small, clean magnetic stir bars were then placed at the base of each chamber. Each chamber was connected to a Bilayer Clamp amplifier (Warner Instruments, BC-635) *via* a pair of Ag/AgCl electrodes immersed in saturated KCl electrolyte. Salt bridges made of 3% agar equilibrated in 3 M KCl were used to connect the electrodes to the solutions in each chamber. The current and voltage outputs were monitored and recorded using Igor-Pro software (Wavemetrics). Syringes (1 ml volume) filled with chamber buffer were connected to each chamber, allowing the solution to be gradually and alternately brought up on either side until it was above the hole in the partition. Lipid bilayer formation across the hole was detected by an increase in capacitance and the appearance of a square current wave on the oscilloscope in response to a triangle wave voltage. If this was not observed, the syringes were used to lower the solution on either side and then to alternately and gradually bring the solution back up on either side until a stable lipid bilayer was formed. Syringes were then removed, a magnetic stirrer was turned on, and the system’s voltage was adjusted as noted in each experiment. pH adjustments were made during each experiment using precalibrated volumes of 0.5 M HCl or 0.5 M KOH. Protein and other compounds necessary for each experiment were added directly to the chamber and to the final concentrations indicated in the figure legend. The “*cis*” chamber is defined as the side that received protein first and mimics the endosome lumen/extracellular space of a cell. The “*trans*” chamber is then defined as the side that mimics the intracellular space of a cell. The reported voltage is that of the *cis* side relative to the *trans*.

It is important to note that all planar bilayer traces in this article reflect macroscopic ion currents, due to the combined currents of many APOL1 channels. The lipid bilayer experiment with the lowest conductance (and fewest channels) is represented in Figure 9*A*. This bilayer contained an absolute minimum of 35 channels (assuming a unitary conductance of ∼20 pS (14)), although the true number is considerably higher owing to the limited probability of channel opening, even at neutral pH. The results are therefore akin to a fluorescence-activated cell sorting plot in that many events are averaged. A comparison is considered significant (*e.g.*, between two different proteins or before and after a treatment) if a difference is observed on the same bilayer and the effect is seen consistently in at least three independent experiments. (Absolute currents can vary markedly between experiments, but relative currents before and after treatment on the same bilayer are consistent.)

### pH-dependent dimerization and silver stain of cysteine-modified APOL1 proteins

Cysteine-modified APOL1 proteins (∼0.159 μM) were incubated in 0.2 ml of buffer (5 mM succinate, 5 mM Hepes, 0.5 mM KCl, 5 mM CaCl_2_) at room temperature under varying pH conditions to facilitate the dimerization process for up to 2 h. The pH was adjusted with 1 M HCl and 1 M NaOH to pH 5.6 or pH 7.4, respectively. After incubation, the proteins were run on an any-KD SDS-PAGE gel (BioRad, 4569034, and Thermo Scientific PageRuler-Plus prestained 10–250 kDa protein ladder, PI26620). Before running on a gel, all pH 5.6 samples were adjusted to pH 7.4 immediately prior to gel loading. The gels were silver stained in accordance with the manufacturer's protocol (Thermo Scientific, 24612).

## Data availability

All relevant data are contained in the article or in the supplementary information.

## Supporting information

This article contains supporting information (14).

## Conflict of interest

The authors declare that they have no conflicts of interest with the contents of this article.
