# Supplementary material for: Coiled-coil binding of the leucine zipper domains of APOL1 is necessary for the open cation channel conformation
Source: J Biol Chem. 2021 Jul 29;297(3):101009. doi: 10.1016/j.jbc.2021.101009 (PMC8446801; doi:10.1016/j.jbc.2021.101009)
Supplement: Supplemental Figures S1–S7 and Tables S1–S2 [file mmc1.pdf]

Coiled-coil binding of the leucine zipper domains of APOL1 is necessary for the open cation channel conformation

Charles Schaub[1,2,3], Penny Lee[1,4], Alisha Racho-Jansen[1], Joe Giovinazzo[1,5], Nada Terra[1,6], Jayne Raper[1,2*] and Russell Thomson[1*]

[1]Department of Biological sciences, Hunter College, City University of New York, USA.
[2]The Ph.D. Program in Biochemistry, The Graduate Center of the City University of New York.

Now at [3]Vanderbilt University, Nashville, Tennessee, USA; [4]John Jay College, City University of New York, USA; [5]University of Colorado School of Medicine, Aurora, Colorado, USA and [6]Icahn School of Medicine at Mount Sinai, New York, USA

*-Corresponding authors

E-mail:
raper@genectr.hunter.cuny.edu
rthomson@genectr.hunter.cuny.edu

Running Title: Molecular determinants of APOL1 cation channel activity

Supplementary Tables S1 and S2; Figures S1-S7

Software	PTM0 (84-101)	PTM1 (174-198)	PTM2 (205-230)	PTM3 (257-276)	PTM4 (335-356)
TMHMM<br>TMHMM		+<br>+	+<br>+		+<br>+
TMPRED<br>TMPRED	 +	+<br>+	+<br>+	 +	+<br>+
DAS<br>DAS	+<br>+	+<br>+	+<br>+		+<br>+
Split Server<br>Split Server		+<br>+	+<br>+		+<br>+
OCTOPUS<br>OCTOPUS		+<br>+	+<br>+		+<br>+
PHOBIUS<br>PHOBIUS		+<br>+	+<br>+		+<br>+
HMMTOP<br>HMMTOP		+<br>+	+<br>+		
JPRED-4<br>JPRED-4		+<br>+	+<br>+	+<br>+	+<br>+
MEMSAT<br>MEMSAT		+<br>+			+<br>+

Table S1. APOL1 G0 transmembrane predictions (aa 28-398). A plus-sign (+) indicates the corresponding prediction model identified a putative transmembrane domain within the specified amino acid range (+/- two amino acid residues). Where applicable (red font), negatively charged aspartate and glutamate residues were replaced for uncharged asparagines and glutamines to simulate the effect of protonation at acidic pH.

Protein	Trypanolytic with MPB	Streptavidin Effect
G58C	Functional	None
I68C	Functional	None
K78C	Functional	None
L88C	Functional	None
V98C	Functional	None
**A108C**	**Functional**	***Cis***
**V244C**	**Functional**	***Cis***
**G278C**	**Functional**	***Trans***
R288C	Functional	None
H298C	Functional	None
R305C	Functional	None
E328C	Functional	None
**K373C**	**Functional**	***Cis***

Table S2. Summary of SCAM results. Functional/trypanolytic MPB-conjugated APOL1 proteins were selected for SCAM analysis. "Functional" denotes Cys-MPB conjugated proteins that reduced trypanosome survival to less than 50% when incubated with trypanosomes at 800 ng/ml for 24 hrs. Channels formed by each protein were then assessed for functional modification by streptavidin as outlined in Figure 7 and demonstrated in Figure 8. Colored text indicates the positioning of each residue within the 4-TM topology model shown in Figure 7 (red: lumenal/extracellular; blue: cytoplasmic).

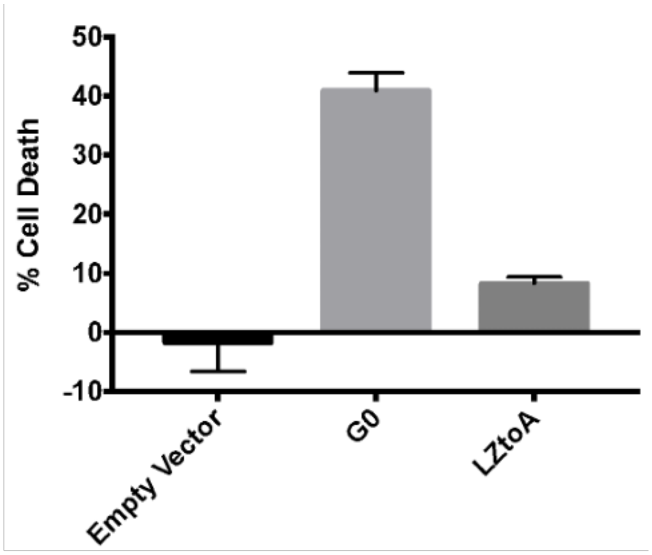

Figure S1. Reduced cytotoxicity of APOL1 LZA compared to APOL1 G0 following overexpression in HEK293 cells. HEK293 cells were plated at 1 x 10^6/well and transiently transfected for 24 hours with expression vector alone (empty vector), or with expression vector containing the APOL1 G0, or APOL1 LZA coding sequences. Percent cell death is determined by the LDH release assay relative to untransfected controls and plotted as mean +/- SD. of triplicate measurements. Note that the LZA mutations resulted in a ~75% reduction in cell death compared to APOL1 G0.

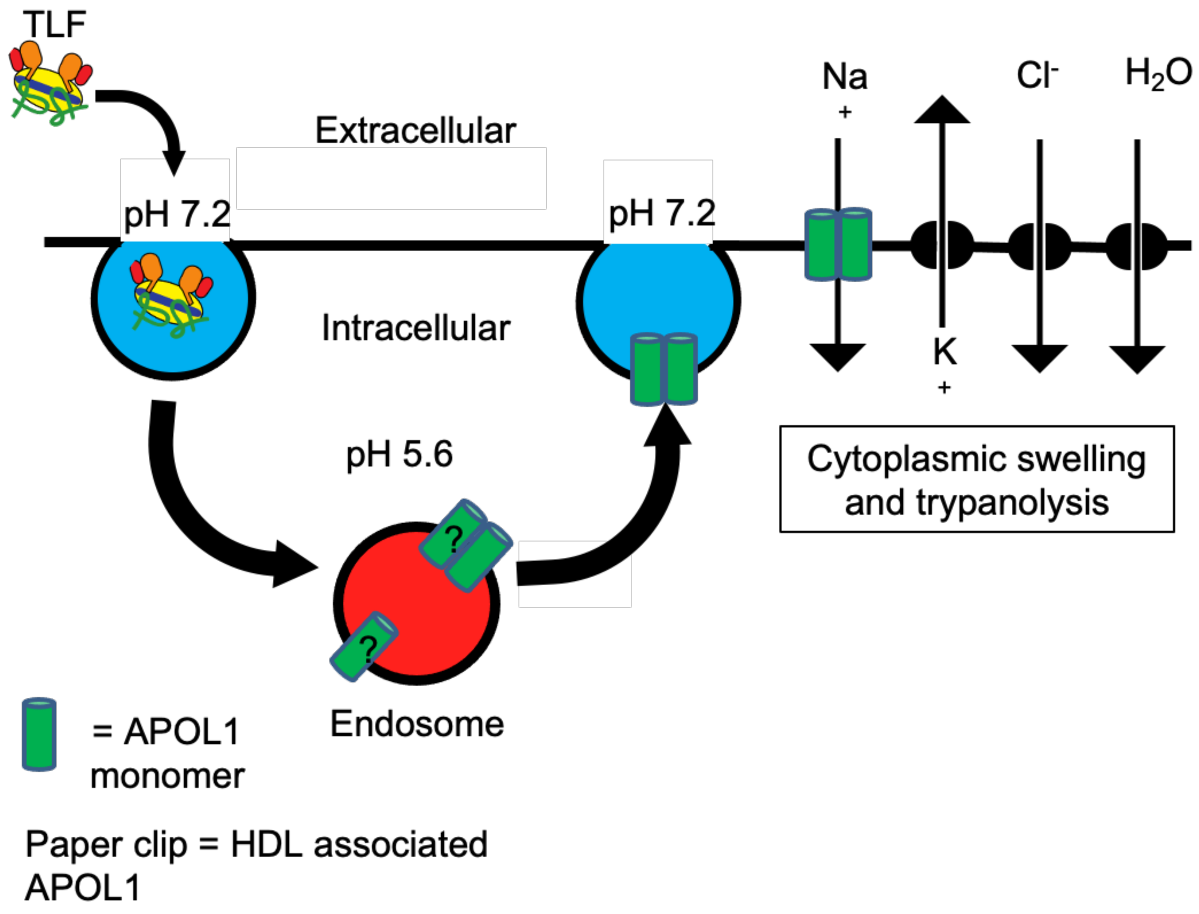

Figure S2. Model of TLF/APOL1 mediated trypanolysis. The TLF complex is taken up by receptor mediated endocytosis via the *Trypanosoma brucei* haptoglobin-hemoglobin receptor (TbHpHbR). Once in the acidic endosome, APOL1 is able to insert into the endosomal membrane. Endocytic recycling then allows inserted APOL1 to be transported to the plasma membrane. At neutral pH, APOL1 channels open, leading to sodium influx accompanied by osmotic swelling and lysis. Adapted from (1).

A

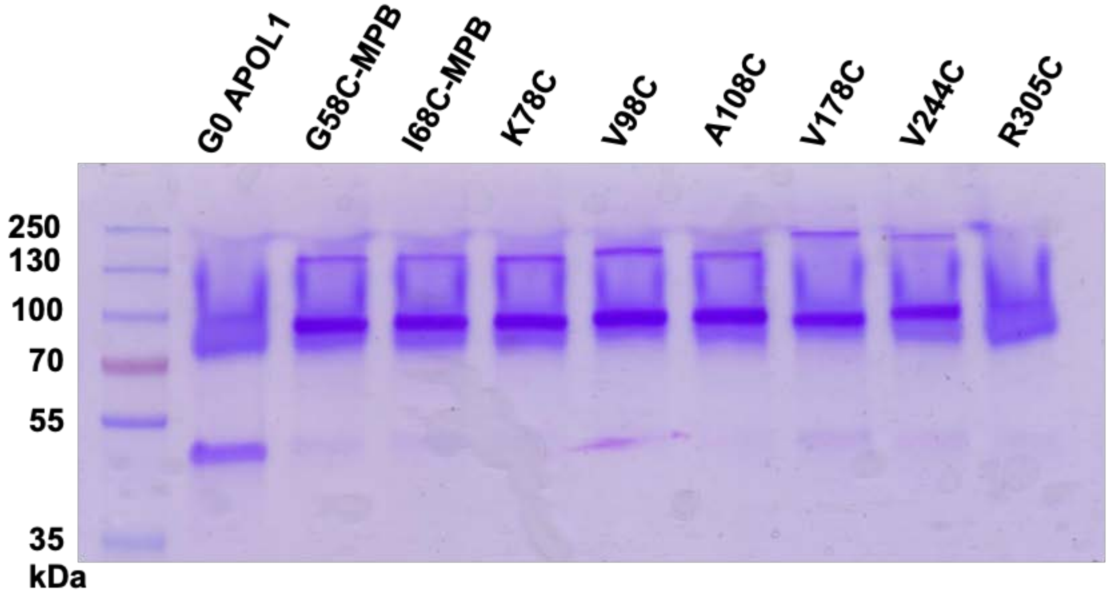

G0 APOL1
G58C-MPB
I68C-MPB
K78C
V98C
A108C
V178C
V244C
R305C
250
130
100
70
55
35
kDa

B

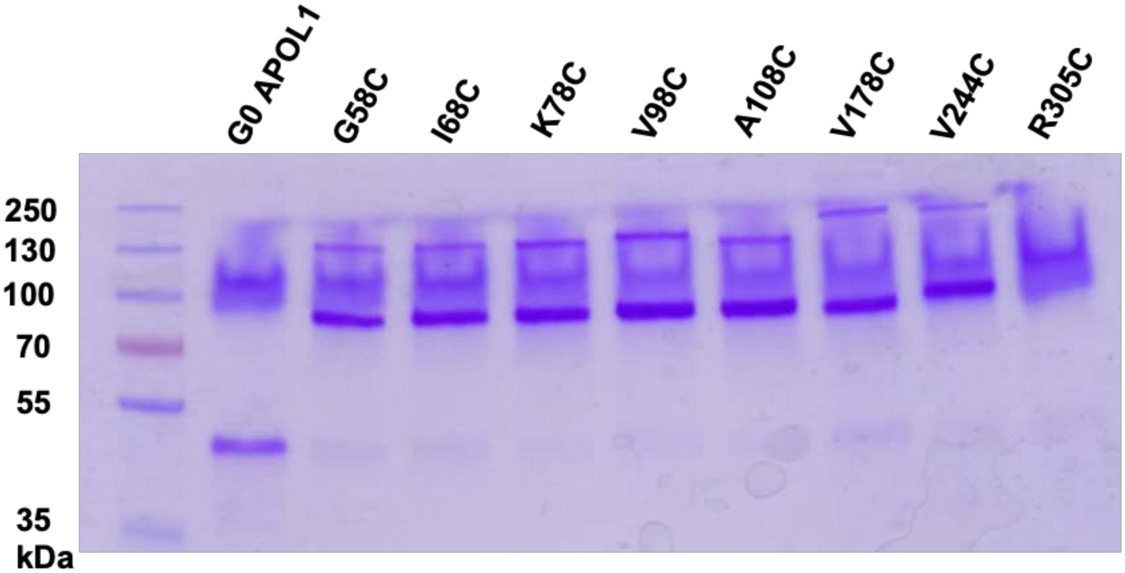

G0 APOL1
G58C
I68C
K78C
V98C
A108C
V178C
V244C
R305C
250
130
100
70
55
35
kDa

Figure S3. Representative conjugation efficiencies of MPB with cysteine-substituted APOL1 proteins. (A) Equivalent concentrations of APOL1 G0 and various MPB-conjugated APOL1 proteins (~0.94 μM) were briefly incubated with ~9.4 μM streptavidin and then separated by non-reducing SDS-PAGE. Indicated above each well is the cysteine residue to which MBP was attached. Coomassie staining reveals a dark APOL1-sized (~43 kDa) band in the APOL1 G0 lane, that was largely absent from the MBP-conjugated proteins, due to the formation of SDS-resistant MBP-streptavidin complexes. **(B)** The same experiment as above, except a reducing SDS-PAGE gel was run and stained with Coomassie. Again, only faint APOL1 sized (~43 kDa) bands can be seen in the APOL1-MPB lanes, showing high labeling efficiency after purification.

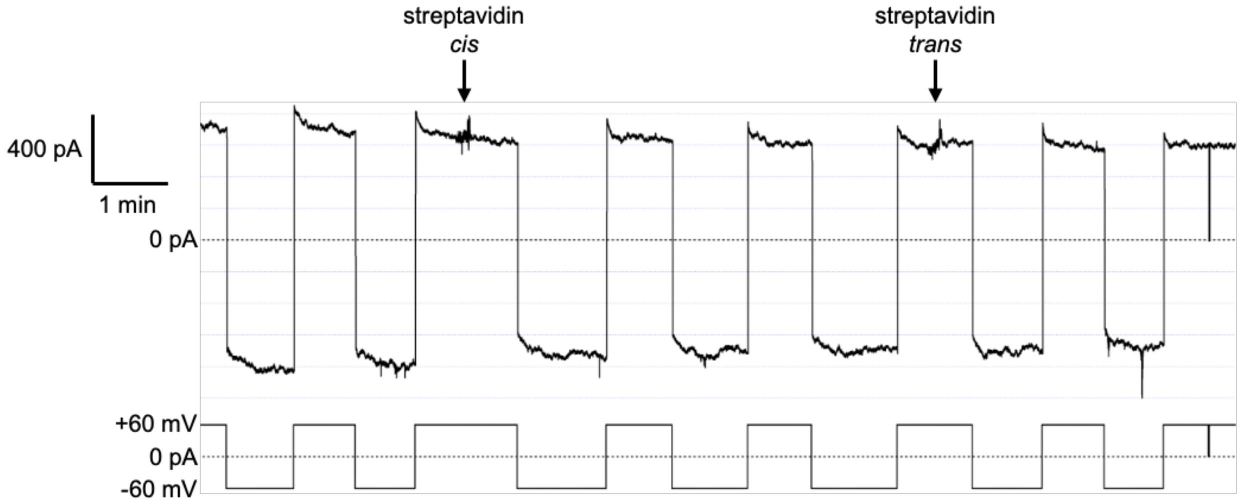

Figure S4. The conductance formed by APOL1 G0 is unaffected by *cis* or *trans* streptavidin. (A) Before the start of the record, APOL1 G0 (24 nM) was added to the bilayer (*cis* pH 6.2, *trans* pH 7.2) until a detectable conductance was obtained, and then the *cis* side was perfused with chamber buffer (pH 6.2). The pH was then adjusted to pH 7.5 to allow for channel opening. The voltage was alternated between +/- 60 mV (lower trace), and the current was recorded as shown (upper trace). As is typical of APOL1, the current magnitude was greater at negative voltage than at positive voltage. This remained true after addition of streptavidin (94 nM) to the *cis* and then *trans* sides as indicated.

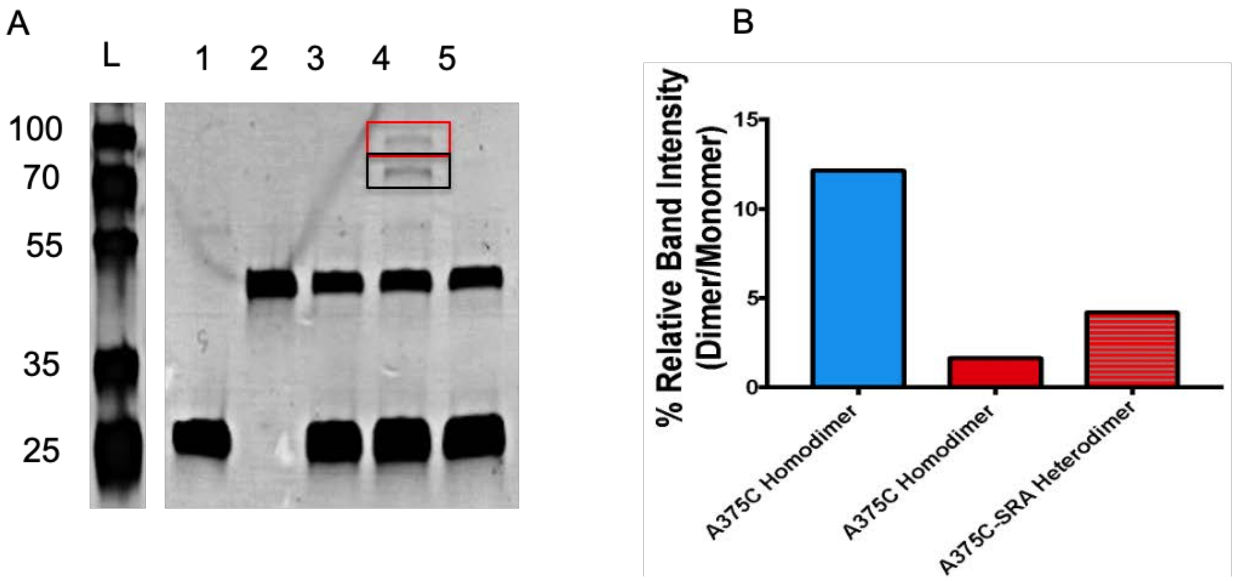

Figure S5. APOL1 A375C forms a disulfide bridges that can be blocked by SRA. **(A)** APOL1 A375C was incubated in bilayer buffer in the presence of a 2-fold molar excess SRA for a total of two hours under the different pH conditions indicated below. The reactions were then separated on a non-reducing SDS-PAGE gel, and silver stained. L – Ladder; Lane 1: SRA, untreated; Lane 2: APOL1 A375C, untreated; Lane 3: APOL1 A375C + SRA, 2 hours pH 7.4; Lane 4: APOL1 A375C + SRA, 1 hour at pH 5.6, followed by 1 hour at pH 7.4; Lane 5: APOL1 A375C + SRA, 2 hours at pH 5.6. In addition to the expected ~90 kDa band APOL1 homodimer band (red box), there was also a ~70 kDa band (black box), potentially resulting from a disulfide-linked APOL1-SRA heterodimer. **(B)** Densitometric analysis comparing relative band intensity of the APOL1 A375C homodimer (~90 kDa band) in Fig. 5D compared to the homodimer (90 kDa band) and heterodimer (70 kDa band) in panel A. Band intensity was determined by pixel density with Jmol software, and the dimer value was normalized to the monomer value.

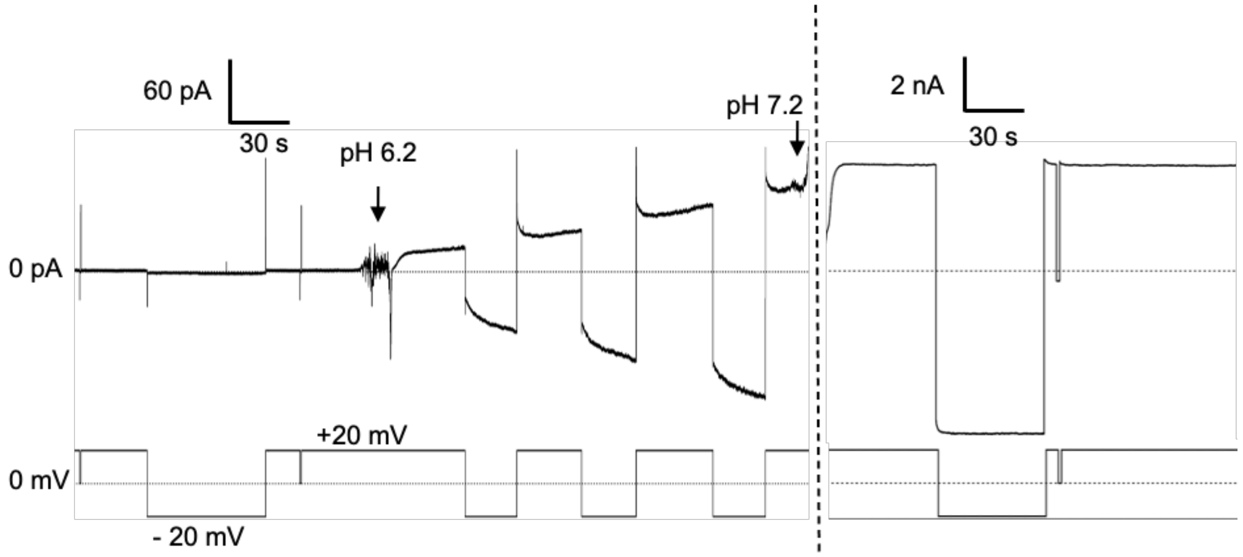

Figure S6. APOL1 associates lipid bilayers as an inactive precursor at pH 6.7. Before the start of the record, and with the cis side buffered at pH 6.7, APOL1 (1 ug) was allowed to bind to the lipid bilayer for several minutes and then the cis side was perfused with bilayer buffer to remove soluble and loosely bound protein. The *cis* side was then adjusted to pH 6.7, followed by pH 6.2 as shown above the upper (current) trace. The voltage was adjusted between +/- 20 mV as shown in the lower trace. Note that after adjustment to pH 6.2 the current magnitude increased slowly and continuously with time, as additional membrane-bound APOL1 was converted from an inactive precursor into a membrane-inserted active channel conformation. As expected, the current magnitude then increased significantly upon *cis* neutralization (pH 7.2), as the pH-gated channels that formed at pH 6.2 were rapidly converted into their maximally open state (note change in current scale at the vertical dashed line). Note that the current magnitude does not continue to increase after neutralization, because channel formation requires acidic pH.

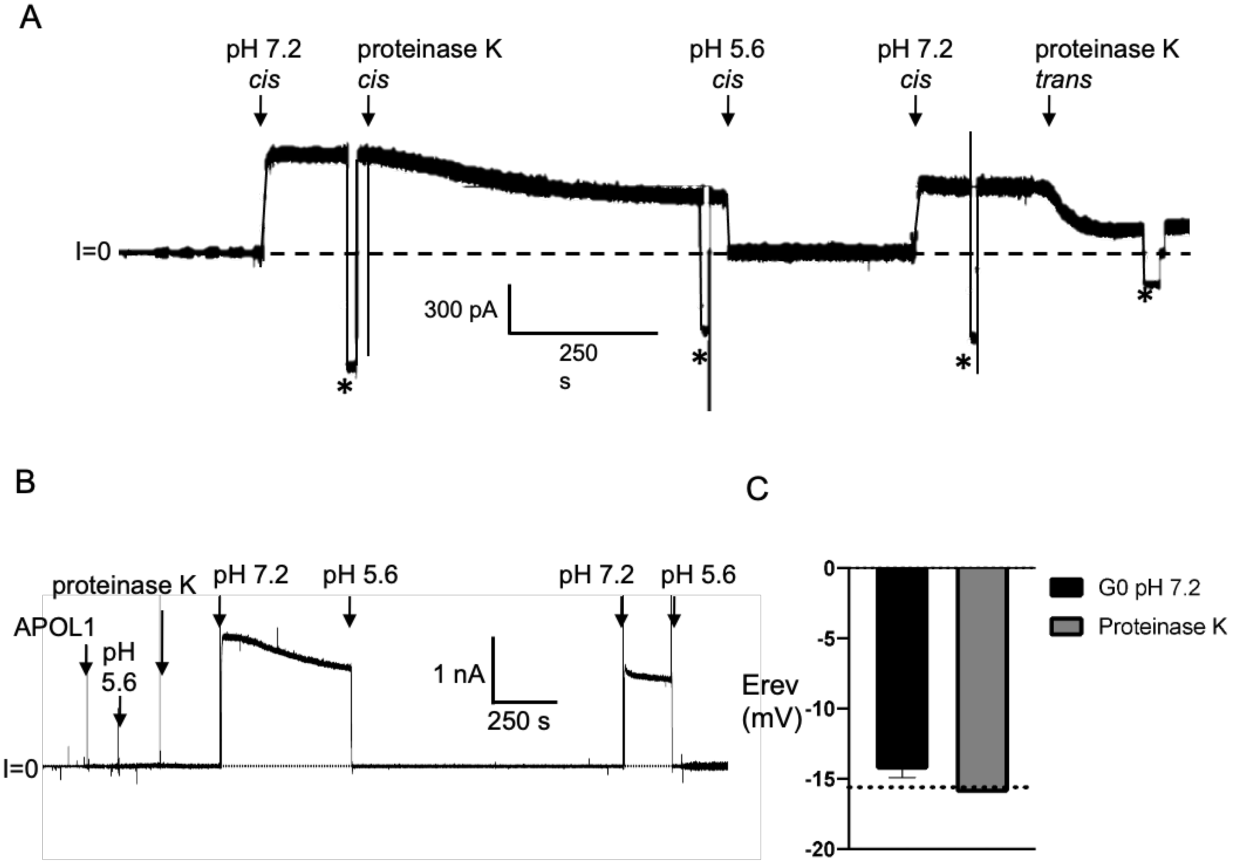

Figure S7. Effect of Proteinase K on the conductance formed by APOL1 in planar lipid bilayers. (A) Before the start of the record, ~360 ng of APOL1 G0 was added to the *cis* chamber at pH 5.4 and allowed sufficient time to insert into the membrane. Then, the *cis* chamber was then neutralized to pH 7.2, as indicated, allowing for channel opening. Proteinase K was then added (1.8 mAU/mL final), which resulted in a decrease in conductance to a plateau at around 50% of the starting level. After the channels were then closed and reopened by the indicated pH changes, Proteinase K was added to the *trans* chamber, which resulted in a similar decrease in conductance. The voltage was held at +20 mV, except for brief excursions to -20 mV, as indicated by an asterisk. (B) With symmetrical pH 7.4 solutions, ~360 ng of APOL1 G0 was added to the *cis* chamber, which was then acidified for 5 minutes to allow for membrane insertion. Proteinase K (1.8 mAU/mL final) was then added with the cis chamber still buffered at pH 5.6 (closed channel state). The cis side was then neutralized which resulted in an immediate increase (due to channel opening), followed by a gradual decline in conductance. Typical pH gating was then confirmed by *cis* acidification and neutralization. The voltage was held at + 20 mV throughout. (C) Reversal potential (E_{rev}) of APOL1 G0 conductance, with (grey) and without (black) *cis* and *trans* treatment with Proteinase K at pH 7.2, with a *cis/trans* KCl activity gradient of 1.7:1. The expected reversal potential, assuming ideal selectivity for potassium, is shown with a dotted black line**.**

<u>**Reference**</u>

1. Thomson, R., and Finkelstein, A. (2015) Human trypanolytic factor APOL1 forms pH-gated cation-selective channels in planar lipid bilayers: relevance to trypanosome lysis. *Proc Natl Acad Sci U S A* **112**, 2894-2899
